# Blood-derived dendritic cell vaccinations induce immune responses that correlate with clinical outcome in patients with chemo-naive castration-resistant prostate cancer

**DOI:** 10.1186/s40425-019-0787-6

**Published:** 2019-11-14

**Authors:** Harm Westdorp, Jeroen H. A. Creemers, Inge M. van Oort, Gerty Schreibelt, Mark A. J. Gorris, Niven Mehra, Michiel Simons, Anna L. de Goede, Michelle M. van Rossum, Alexandra J. Croockewit, Carl G. Figdor, J. Alfred Witjes, Erik H. J. G. Aarntzen, Roel D. M. Mus, Mareke Brüning, Katja Petry, Martin Gotthardt, Jelle O. Barentsz, I. Jolanda M. de Vries, Winald R. Gerritsen

**Affiliations:** 1 Department of Tumor Immunology and Medical Oncology, Radboud Institute for Molecular Life Sciences, Radboudumc, Geert Grooteplein 26, 6525 GA Nijmegen, The Netherlands; 2 Department of Medical Oncology, Radboudumc, Nijmegen, The Netherlands; 3 Department of Urology, Radboudumc, Nijmegen, The Netherlands; 4 Department of Pathology, Radboudumc, Nijmegen, The Netherlands; 5 Department of Pharmacy, Radboudumc, Nijmegen, The Netherlands; 6 Department of Dermatology, Radboudumc, Nijmegen, The Netherlands; 7 Department of Hematology, Radboudumc, Nijmegen, The Netherlands; 8 Department of Radiology and Nuclear Medicine, Radboudumc, Nijmegen, The Netherlands; 9 Miltenyi Biotec GmbH, Bergisch Gladbach, Germany

**Keywords:** Castration-resistant prostate cancer, Dendritic cell vaccination, Immunotherapy

## Abstract

**Background:**

Clinical benefit of cellular immunotherapy has been shown in patients with castration-resistant prostate cancer (CRPC)**.** We investigated the immunological response and clinical outcome of vaccination with blood-derived CD1c^+^ myeloid dendritic cells (mDCs; cDC2) and plasmacytoid DCs (pDCs).

**Methods:**

In this randomized phase IIa trial**,** 21 chemo-naive CRPC patients received maximally 9 vaccinations with mature mDCs, pDCs or a combination of mDCs plus pDCs. DCs were stimulated with protamine/mRNA and loaded with tumor-associated antigens NY-ESO-1, MAGE-C2 and MUC1. Primary endpoint was the immunological response after DC vaccination, which was monitored in peripheral blood and in T cell cultures of biopsies of post-treatment delayed-type hypersensitivity-skin tests. Main secondary endpoints were safety, feasibility, radiological PFS (rPFS) and overall survival. Radiological responses were assessed by MRIs and contrast-enhanced ^68^Ga-prostate-specific membrane antigen PET/CT, according to RECIST 1.1, PCWG2 criteria and immune-related response criteria.

**Results:**

Both tetramer/dextramer-positive (dm^+^) and IFN-γ-producing (IFN-γ^+^) antigen specific T cells were detected more frequently in skin biopsies of patients with radiological non-progressive disease (5/13 patients; 38%) compared to patients with progressive disease (0/8 patients; 0%). In these patients with vaccination enhanced dm^+^ and IFN-γ^+^ antigen-specific T cells median rPFS was 18.8 months (*n* = 5) vs. 5.1 months (*n* = 16) in patients without IFN-γ-producing antigen-specific T cells (*p* = 0.02). The overall median rPFS was 9.5 months. All DC vaccines were well tolerated with grade 1–2 toxicity.

**Conclusions:**

Immunotherapy with blood-derived DC subsets was feasible and safe and induced functional antigen-specific T cells. The presence of functional antigen-specific T cells correlated with an improved clinical outcome.

**Trial registration:**

ClinicalTrials.gov identifier NCT02692976, registered 26 February 2016, retrospectively registered.

## Background

Prostate cancer (PCa) remains the most common non-cutaneous malignancy and the second leading cause of cancer-related death in men [1]. For years, docetaxel-based chemotherapy was the only effective treatment for castration-resistant prostate cancer (CRPC) [2–4]. This changed with the approval of multiple agents, including androgen-signaling-targeted inhibitors abiraterone and enzalutamide [5–8], the cell-based vaccine sipuleucel-T [9], the radionuclide radium-223 [10] and second-line taxane cabazitaxel [11, 12]. These new agents extend overall survival (OS) with approximately 3–4 months [5–10, 13].

Recent advances in the field of cancer immunotherapy led to growing interest in prostate cancer immunotherapy. Immune-checkpoint inhibitor ipilimumab failed to show survival benefit in advanced PCa in phase III trials [14, 15]. Sipuleucel-T is still the only FDA-approved cellular immunotherapy for men with minimally symptomatic metastatic CRPC [9]. In Europe, sipuleucel-T is not available since its marketing authorization was withdrawn in 2015 at the request of the manufacturer [16]. Sipuleucel-T is an autologous antigen-presenting cell-based vaccination strategy, targeting prostatic acid phosphatase on prostate adenocarcinomas. The proposed mechanism of sipuleucel-T is induction of antigen-specific immune responses against PCa cells [17]. However, a complete understanding of the mechanism of action of sipuleucel-T is lacking. It remains unclear whether sipuleucel-T acts via priming of naive T cells through antigen presentation since the sipuleucel-T products contained more than 60% CD3^+^ T cells and < 20% cells expressing the co-stimulatory molecule CD54, indicated as dendritic cells (DCs) [18]. It remains unclear whether sipuleucel-T harbors mature DC properties necessary for priming of naive T cells. Therefore, vaccination with antigen-specific blood-derived DCs may be a more potent alternative.

DCs are the most potent antigen-presenting cells of the immune system. They are crucial for inducing adaptive immune responses [19] and are widely studied in clinical trials, predominantly in advanced melanoma patients [20–24]. Antigen-loaded autologous DCs are given to patients with the intention of inducing functional tumor-associated antigen (TAA)-specific T cell responses. There are two major types of naturally occurring DCs that circulate in the blood [25], myeloid DCs (mDCs) and plasmacytoid DCs (pDCs). These subsets can be distinguished by the presence of different surface markers. mDCs can be further subdivided into two populations, based on their differential surface expression of CD1c (BDCA-1; cDC2) and CD141 (BDCA-3; cDC1) [25]. mDCs act in particular against bacteria [26] and have the capacity to prime cytotoxic T cell responses [27]. pDCs produce high amounts of type I interferons, mainly in response to viral stimuli [28, 29].

mDCs and pDCs express different pattern recognition receptors, respond differently to stimuli and have different migration patterns [30]. This suggests that mDCs and pDCs have unique functional characteristics and may act synergistically by bi-directional crosstalk between the subsets and T cells [28, 30, 31]. Previously, we studied the safety, immunogenicity and clinical efficacy of pDC and CD1c^+^ mDC vaccinations in stage IV melanoma patients [23, 24]. In these studies, promising tumor-specific T cell responses, cytokine production profiles and clinical responses were observed. This supports the use of both pDCs and cDC2 for evaluation in a phase IIa clinical trial in patients with CRPC .

## Materials and methods

### Patients

In this open-label, randomized, phase IIa study we screened 44 chemotherapy-naive patients with CRPC. Patients with rising prostate-specific antigen (PSA) were closely monitored to detect early biochemical progression. Patients were screened for study eligibility as soon as patients met the criteria for CRPC [32]. Since there is no clear consensus om the correct timing for CRPC treatment, this window was used to screen asymptomatic or minimally symptomatic CRPC patients. Twenty-two of the screened patients were HLA-A-*0201. One of these patients was excluded because a second primary malignancy was detected (Additional file 1: Figure S1). All 21 included patients had histologically confirmed adenocarcinoma of the prostate. Eligible patients had not received any immunotherapy, docetaxel, cabazitaxel or treatment with the RANKL-inhibitor denosumab. Concurrent use of glucocorticoids up to 10 mg per day or a prednisone equivalent was permitted. Patients requiring opioids for cancer-related pain at screening were excluded. Patients had no visceral metastases. Other eligibility criteria were: an Eastern Cooperative Oncology Group (ECOG) performance status grade of 0 or 1; ongoing luteinizing hormone-releasing analogue therapy or status after bilateral orchidectomy; serum testosterone level of < 1.73/l (< 50 ng/dl); absence of active autoimmune diseases, absence of an active viral infection or allergy to shell fish; and no significant laboratory abnormalities (hemoglobin > 5.6 mmol/l (9.0 g/dl); white blood cell count of > 3.0 × 10^9^/l; platelets > 100 × 10^9^/l; serum creatinine < 150 μmol/l; AST/ALT < 3 x ULN, and serum bilirubin < 25 μmol/l, exception Gilbert’s syndrome). Baseline disease sites were assessed using ^68^Ga- prostate-specific membrane antigen (PSMA) PET/CT scans [33], including thin-section diagnostic CT (3 mm) and ferumoxtran-10-enhanced MRIs [34, 35] and regular MRI of bones and lymph nodes. Response evaluation was assessed according to Response Evaluation Criteria In Solid Tumours (RECIST) version 1.1 [36] and the Prostate Cancer Clinical Trials Working Group 2 (PCWG2) criteria [37]. The immune-related response criteria and the iRECIST criteria were used to assess immune unconfirmed progressive disease [38–41]. Response evaluation was assessed by using contrast-enhanced ^68^Ga-PSMA PET/CT scans and ferumoxtran-10-enhanced MRIs at 3 months, and for patients with long-term clinical benefit after 12 and 24 months. Regular follow-up MRI of lymph nodes and bones was performed at 6, 9, 15, 18 and 21 months. Measurable lesions were measured in at least one dimension with longest diameter ≥ 10 mm. Small lesions (longest diameter < 10 mm or pathological lymph nodes with < 15 mm short axis) are considered non-measurable disease. Bone metastases were documented and assessed according to PCWG2 criteria. Patients with absence of disease progression, defined as patients with a radiological complete or partial response or stable disease for > 6 months, were eligible for a maintenance cycle of three biweekly vaccinations. Patients without progressive disease after 12 months were eligible for a final vaccination cycle (Additional file 2: Figure S2A). Baseline characteristics and prior therapies are presented in Table 1.

**Table 1:** Baseline characteristics of patients treated with blood-derived DC vaccinations

Patient^a^	Age (years)	Gleason score	Prior treatments	Time start ADT to CRPC (mo)	Baseline PSA (ug/l)^b^	Baseline LDH (U/l)^b^	Baseline ALP (U/l)^b^	All disease sites at baseline^c^
mDC-01	60	4 + 5	GOS, BIC	55.4	5.8	187	100	Local, LN, bone
mDC-02	67	4 + 4	PLND, GOS	20.7	4.6	152	80	Local, LN
mDC-03	67	3 + 4	PRTX, GOS, BIC	93.9	10	257	61	Local, LN
mDC-04	60	4 + 3	RP, SRTX, PLND, GOS, BIC	21.5	39	147	113	LN, bone
mDC-05	78	3 + 4	RP, SRTX, PLND, SO, BIC	34.7	4.8	211	79	LN
mDC-06	71	4 + 5	BIC + DUT, SO	94.5	40	185	101	Local, LN, bone
mDC-07	72	5 + 5	CRT, PRTX, PLND, GOS, BIC	27.7	260	222	260	LN, bone
pDC-01	72	5 + 5	PRTX, PLND, LEU, BIC	28.5	6.3	181	98	LN
pDC-02	59	4 + 5	PRTX, GOS, BIC, NIL, DUT, ABI + P/D	83.4	3.6	222	102	Local, LN, bone
pDC-03	59	5 + 5	PLND, LEU, BIC	29.0	8.4	176	81	Local, LN, bone
pDC-04	70	3 + 4	RP, SRTX, PLND, BIC, SO	42.3	19	174	88	LN
pDC-05	65	4 + 3	RP, SRTX, LEU, BIC	41.6	2.6	169	138	LN, bone
pDC-06	79	5 + 4	GOS	85.7	5.7	164	95	Local, LN
pDC-07	82	4 + 5	GOS, BIC	91.6	17	201	53	Local, LN, bone
combiDC-01	74	3 + 4	GOS	46.1	38	168	96	Bone
combiDC-02	67	4 + 4	LEU, BIC	27.6	41	172	83	Local, bone
combiDC-03	63	5 + 4	GOS, LEU, BIC	53.9	18	233	79	Local, LN
combiDC-04	73	4 + 3	LEU, BIC, ENZ	39.3	8.7	179	73	Local, bone
combiDC-05	61	4 + 5	GOS, BIC, ENZ	40.5	3.7	205	123	Local, LN, bone
combiDC-06	73	4 + 4	PRTX, GOS, LEU, BIC, ENZ	20.4	11	131	73	LN, bone
combiDC-07	53	4 + 4	DEG, BIC, ABI + P, SO, ARN-509	29.4	120	161	83	LN, bone

ABI + P: abiraterone plus prednisone; ABI + P/D: abiraterone plus prednisone + switch of prednisone to dexamethasone at PSA-progression; ADT: androgen deprivation therapy; ALP: alkaline phosphatase; ARN-509: apalutamide, ClinicalTrials.gov identifier (NCT number): NCT01946204; *CRT* cryotherapy, *BIC* bicalutamide, *CRPC* castration-resistant prostate cancer, *DC* dendritic cells, *DEG* degarelix, *DUT* dutasteride, *ENZ* enzalutamide, *GOS* goserelin, *LDH* lactate dehydrogenase, *LEU* leuprorelin, *LN* lymph node, *mo* months, *NIL* nilutamide, *PLND* pelvic lymph node dissection, *PRTX* primary radiotherapy, *PSA* prostate-specific antigen, *RP* radical prostatectomy, *SO* surgical orchidectomy, *SRTX* salvage radiotherapy

^a^vaccination with myeloid DC (patient mDC-01 to mDC-07), plasmacytoid DC (patient pDC-01 to pDC-07) or combined myeloid DC + plasmacytoid DC (patient combiDC-01 to combiDC-07)

^b^measured prior to apheresis

^c^attributed by experienced nuclear medicine specialists and radiologists. Detected at advanced imaging with contrast enhanced ^68^Ga-prostate-specific membrane antigen PET/CT scans, ferumoxtran-10-enhanced MRIs and MRI bones using Response Evaluation Criteria In Solid Tumors (RECIST) 1.1 and Prostate Cancer Clinical Trials Working Group 2 (PCWG2) criteria

### Study design and objectives

Patients with CRPC were randomly assigned in a 1:1:1 ratio to receive CD1c^+^ mDC vaccinations (2–5 × 10^6^ cells per injection; arm A), pDC vaccinations (1–3 × 10^6^ cells; arm B), or combined CD1c^+^ mDC and pDC vaccinations (combiDC; 3–8 × 10^6^ cells; arm C). One cycle of vaccinations consisted of three biweekly vaccinations administered intranodally in a clinically tumor-free lymph node by our expert radiologist or nuclear medicine physician. One to two weeks after the third vaccination a delayed-type hypersensitivity (DTH)-skin test was performed after intradermal administration of 1–10 × 10^5^ cells [42]. Adverse events were defined in accordance with the Common Terminology Criteria for Adverse Events (CTCAE) version 4.0. Primary endpoint of the study was the immunological response after DC vaccinations. Secondary objectives were safety, feasibility, quality of life and clinical efficacy (radiological progression-free survival (rPFS), OS, prostate-specific antigen doubling time (PSAdt), time to opiate use for cancer-related pain, time to SRE, time to decline in WHO/ECOG performance score by ≥1 point and time to the initiation of docetaxel chemotherapy). rPFS was defined as the time from apheresis to radiological progression of soft-tissue lesions or two or more new bone lesions or death from any cause. The event date of the unconfirmed progression was used for calculation of rPFS. OS was defined as the time from apheresis to death from any cause. The PSAdt was calculated according to the Memorial Sloan-Kettering Cancer Center guidelines (http://nomograms.mskcc.org/Prostate/PsaDoublingTime.aspx). An SRE was defined as a pathologic fracture, palliative radiotherapy to a bone lesion, spinal-cord compression or surgery involving bone.

### Statistical analysis

Paired t-tests were performed to evaluate immunological responses before and after vaccination and independent-samples t-tests (Mann-Whitney U tests) were used to evaluate differences between groups. Statistical significance was defined as *p* < 0.05 (two-tailed significance level). Time-to-event data were evaluated using the Kaplan-Meier method. Statistical significance was evaluated using the two-sided log-rank test and was defined as *p* < 0.05. Differences between treatment arms were evaluated using one-way ANOVA. Statistical analysis was performed using SPSS® Statistics version 22 software (SPSS Inc., Chicago, IL, USA) and GraphPad Prism 5.03 (GraphPad Software, Inc., San Diego, CA, USA).

### Vaccine preparation and features

CD1c^+^ mDCs and pDCs were manufactured according to Good Manufacturing Practices (GMP). DCs were directly isolated from apheresis products using the fully automated and enclosed immunomagnetic CliniMACS Prodigy isolation system (Miltenyi Biotec, Bergisch-Gladbach, Germany). GMP-grade magnetic bead-coupled antibodies were used, following the manufacturer’s guidelines. For mDC isolation, first, CD19^+^ and CD14^+^ cells were depleted, followed by positive selection of BDCA1^+^ cells with biotin-coated CD1c (BDCA-1) antibodies and anti-biotin coated magnetic beads (arm A). PDC were selected with anti-CD304 (BDCA-4) coupled beads (arm B). When patients were randomized for vaccination with both mDCs and pDCs (arm C), first pDC were selected with anti-CD304 coupled beads, followed by depletion of CD19^+^ and CD14^+^ cells and positive selection of CD1c^+^ cells. mDCs were cultured overnight at a concentration of 1.5 × 10^6^ cells/ml with 800 IU/ml recombinant human GM-CSF in TexMACS GMP medium (both Miltenyi Biotec) supplemented with 2% human serum (Sanquin) and 10 μg/ml keyhole limpet hemocyan (KLH; Immucothel, Biosyn Arzneimittel GmbH) for immunomonitoring purposes. pDCs were cultured overnight at a concentration of 1.5 × 10^6^ cells/ml with 10 ng/ml recombinant human IL-3 in TexMACS GMP medium (both Milteny Biotec) supplemented with 2% pooled human serum. mDCs and pDCs were loaded with HLA-A*0201 binding peptides of NY-ESO-1:157–165 (SLLMWITQC) and MAGE-C2:336–344 (ALKDVEERV) [43] as well as NY-ESO-1 and MUC1 PepTivators (Miltenyi Biotec, Bergisch-Gladbach, Germany)) at a concentration of 1 μM. PepTivators consist of overlapping long peptides that cover the complete protein and bind multiple HLA-types, both MHC class I and II (Additional file 2: Figure S2B).

NY-ESO-1 and MUC1 PepTivators were added during overnight culturing. Thereafter, mDCs and pDCs were activated with premixed protamine HCl (Meda Pharma) and mRNA (gp100, Universitätsklinik Erlangen) for 6 h. Premix ratio 10 μg protamine + 5 μg mRNA, 10 μl premix per ml cell suspension [44]. During the last 3 h of maturation NY-ESO-1 and MAGE-C2 peptides were added at final concentration of 1 μM. This isolation and culture procedure gave rise to mature mDC and pDC meeting the release criteria: sterile, endotoxin level < 7 EU/ml, more than 50% viability, more than 50% purity, expression of CD80 > 50% on pDCs and expression of CD83 > 50% on mDCs. Expression of MHC class I, MHC class II, CD86 and CCR7 was reported, but no release criterium (Additional file 3: Figure S3A-D). Protamine/mRNA complex activated both mDCs and pDCs into mature functional DCs that secrete IFN-α, TNF-α (pDCs only), IL-12p70, and IL-6 (both mDCs and pDCs, but mainly pDCs) (Additional file 3: Figure S3E). Cells were frozen in TexMACS medium containing 10% dimethyl sulfoxide (DMSO; WAK Chemie Medical GmbH) and 40% Albuman (Sanquin), stored at < − 80 °C for max. 2 years and thawed on the day of vaccination. For combined pDC and mDC vaccines, both subsets were pooled in one syringe after thawing.

After apheresis, sufficient amounts of blood-derived DCs could be obtained for at least one vaccination cycle. In two patients randomized for treatment with combiDCs the final CD1c^+^ mDC product did not fulfill the release criteria. Therefore, these patients were vaccinated with pDCs only. Because the primary endpoint of the study was immunological, two extra patients were randomized within the combination arm. In patient pDC-06 the pDC purity was initially only 43%, which increased to 54% after overnight culture and maturation. In patient combiDC-06 CD1c^+^ mDC purity was 41% and thus out-of-specification. Nevertheless, the product was released and administered after accounting for the lower purity by administering at least 2.4 × 10^6^ cells. Consequently, patient received at least the minimum required dose of 2 × 10^6^ CD1c^+^ mDCs per vaccination.

### Flow cytometry

Purity and phenotype of mDCs and pDCs after CliniMACS isolation were determined by flow cytometry with a FACSVerse (BD Biosciences, San Jose, CA, USA) or MACS Quant (Miltenyi Biotec). The following primary monoclonal antibodies and the appropriate isotype or fluorescence minus one controls were used: anti-CD1c-Viobright FITC, anti-BDCA-2-PE, anti-CD20-PE-Vio770, anti-CD123-APC, anti-CD45-APC-Vio770, anti-CD14-VioGreen, anti-FcεRI-VioBlue, anti-CD14-FITC, anti-CD15-PE, anti-CD56-APC, anti-CD3-BioBlue, anti-HLA-ABC-APC, anti-HLA-DR,DP,DQ-APC, anti-CCR7-APC, anti-CD80-APC, anti-CD83-APC and anti-CD86-APC (all Miltenyi Biotec).

### Skin-test infiltrating lymphocyte culture and PBMC analyses

DTH challenges were performed 2 weeks after each vaccination cycle to assess TAA-specific immune response in DC vaccinated patients [42, 45]. DCs used for the DTH-skin test were produced accordingly to the vaccinated cells, except that no KLH was added to the culture medium. At four different sites at the patient’s back maximally 5.0 × 10^5^ peptide-loaded blood-derived DCs were injected intradermally. After 48 h, 6 mm punch biopsies were taken. The biopsies were manually cut and half of the tissue was stored at − 150 °C; the other half was cultured as described previously [42]. After 2 to 4 weeks of culturing, skin-test infiltrating lymphocytes (SKILs) were tested for the presence of tumor antigen-specific T cells. SKILs and peripheral blood mononuclear cells (PBMCs) were stained with 1) anti-CD8-FITC and tetrameric PE- and APC-coupled MHC complexes containing NY-ESO-1 (SLLMWITQC), MAGE-C2 (ALKDVEERV) and MUC1 (LLLLTVLTV) HLA-A*0201 epitopes (all Sanquin, Amsterdam, The Netherlands); or 2) anti-CD8-BV421, anti-CD19-FITC and dextrameric PE- and APC-coupled MHC complexes containing the indicated epitopes (all Immudex, Copenhagen, Denmark). Dextrameric HLA-B*0801 (AAKGRGAAL) and tetrameric and dextrameric HIV (SLYNTVATL) were used as a negative control. Cells were analyzed by flow cytometry. To test peptide recognition, SKILs were challenged with autologous PBMCs loaded with the indicated peptides and PepTivators, **phorbol myristate acetate** (positive control), carcinoembryonic antigen peptide or no peptide (both negative control). Production of interferon-γ (IFN-γ), IL-2, IL-5 and IL-10 was measured in the supernatants after overnight co-culture by cytometric bead array according to the manufacturer’s instructions (BD Biosciences).

### Proliferative and humoral response to KLH

Cellular responses against KLH were measured in a proliferation assay. PBMCs were isolated from blood samples after each vaccination. 1 × 10^5^ PBMCs were plated per well of a 96-well tissue culture microplate either in the presence or absence of KLH. After 4 days of culture, 1 μCi/well of tritiated thymidine was added, incorporation of tritiated thymidine was measured in a beta-counter. A proliferation index (proliferation with KLH/proliferation without KLH) of > 2 was considered positive. Antibodies against KLH were measured in the serum of DC vaccinated patients by ELISA. KLH antibodies were detected with mouse anti-human IgG, IgA, or IgM antibodies labeled with horseradish peroxidase. 3,3′,5,5′-Tetramethylbenzidine was used as a substrate. Plates were measured with a microtiter plate reader at 450 nm. An isotype-specific calibration curve for the KLH response was included in each microtiter plate.

### Immunohistochemistry

Formalin-fixed, paraffin-embedded tissue blocks of prostate biopsies or radical prostatectomy at time of diagnosis were collected from primary treatment centers located in the Netherlands and sections of 4-μm thickness were cut. Slides were deparaffinized using xylene and rehydrated with ethanol. Antigen retrieval was performed by boiling in EnVision™ FLEX target retrieval solution (pH 9, K8004, Dako) for 10 min for MUC1 staining or in Citrate buffer (pH 6, CBB999, ScyTek Laboratories) for 15 min for NY-ESO-1 and MAGE-C2 staining. After cooling down, endogenous peroxidase was blocked using 3% hydrogen peroxidase (76,051,800.1000, EMD Millipore) in PBS (4391.9010, Klinipath). Primary antibodies MUC1 (M0613, clone E26, Dako, dilution: 1/250), NY-ESO-1 (MABC1151, clone D8.38, Merck, dilution: 1/200) and MAGE-C2 (HPA062230, rabbit polyclonal, Merck, dilution: 1/200) were diluted in Normal Antibody diluent (VWRKBD09–999, Immunologic) and were incubated at room temperature for 1 h. Slides were washed between steps with EnVision™ FLEX Wash Buffer (DM831, Dako). Next, incubation with BrightVision poly-HRP-anti-Ms/Rb/Rt IgG (DPVO999HRP, ImmunoLogic) was performed at room temperature for 30 min. Visualization was performed with EnVision™ FLEX DAB Buffered Substrate and EnVision™ FLEX Substrate Buffer (K5207 and SM803; DAKO) for 7 min at room temperature. After dehydration, slides were counterstained with hematoxylin and were enclosed with Quick-D mounting medium (7281, Klinipath). Observed staining was cytoplasmic. Immunoreactivity was evaluated by a pathologist using a semi-quantitative, stepwise scoring system: negative (0% of cells stained), weak (1–10% of cells stained), moderate (11–50% of cells stained) and strong (51 to 100% of cells stained). Representative slides were scanned using the PerkinElmer Vectra (Vectra 3.0.4, PerkinElmer). Testicular or tonsil tissue (positive control) was used for antibody validation (Additional file 4: Figure S4).

## Results

### Patient characteristics

In this prospective study twenty-one eligible patients with CRPC were enrolled. Participants were treated with blood-derived DC vaccines from November 2015 until May 2018. Baseline demographic, disease characteristics and prior therapies for hormone-sensitive PCa and CRPC are listed in Table 1. The described results are based on the cut-off date of 6th of March 2019. The median follow-up is 27.2 months (range 10.7–41.2* months). All twenty-one patients, seven per arm, received at least one cycle of three biweekly DC vaccinations and a DTH-skin test. Thirteen patients also received a second cycle and seven patients a third vaccination cycle.

#### Safety and adverse events

DC vaccinations were well tolerated. In all vaccinated patients only low-grade toxicity (CTCAE grade 1–2) was noticed. Most frequent grade 1–2 toxicity included flu-like symptoms, fatigue, upper respiratory infections, dizziness, vaccination-induced hematomas and injection site reactions. Also, some low-grade laboratory adverse events were seen (Table 2).

**Table 2:** Adverse events

	Vaccinated patients (*n* = 21)^a^	
	Grade 1–2	Grade ≥ 3
Reported toxicity (CTCAE 4.0)		
Any toxicity	21	0
Anemia	15	0
Flu-like symptoms^b^	10	0
Hypoalbuminemia	10	0
Fatigue^c^	8	0
Upper respiratory infection	4	0
Dizziness	3	0
Hematoma	3	0
Lymphopenia	3	0
Hypophosphatemia	3	0
Injection site reaction	2	0
Fever	2	0
Headache^d^	2	0
AST	2	0
Others^e^	1	0

*AST* aspartate aminotransferase, *CTCAE 4.0* Common Terminology Criteria for Adverse Events version 4.0

^a^attributed by investigators

^b^flu-like symptoms include fever, fatigue, chills, body aches, malaise, loss of appetite and headache

^c^fatigue was mentioned separately when it lasted at least 1 day longer than the other flu-like symptoms or when it was present without the other flu-like symptoms

^d^headache was mentioned separately when it was present apart from flu-like symptoms

^e^others include nausea (5%), vomiting (5%), diarrhea (5%), neutropenia (5%), eosinophilia (5%), thrombocytopenia (5%), increased bilirubine (5%) and gamma-glutamyltransferase (5%)

#### Cellular and humoral responses to KLH

mDCs (arm A and C) were loaded with KLH as a control antigen. Since pDCs cannot take up KLH-protein, pDCs were not cultured in the presence of KLH [46]. None of the patients had a KLH-specific proliferation index > 2 at baseline. KLH-specific proliferation increased significantly after one vaccination cycle. In 5 of 7 mDC-treated patients (*p* = 0.01) and 3 of 7 patients in the combiDC group (*p* = 0.04), a T cell response against KLH was observed (Additional file 5: Figure S5A). This indicates that KLH-exposed DCs were indeed able to induce de novo T cell responses to KLH. Humoral responses to KLH were determined in serum before treatment and after each cycle of vaccinations. A significant increase in total IgG titer was seen in mDC vaccinated patients (arm A and C) (Additional file 5: Figure S5B). There was no significant induction of IgA and IgM.

#### Tumor antigen-specific responses in DTH skin-test and blood

**Fig. 1:**
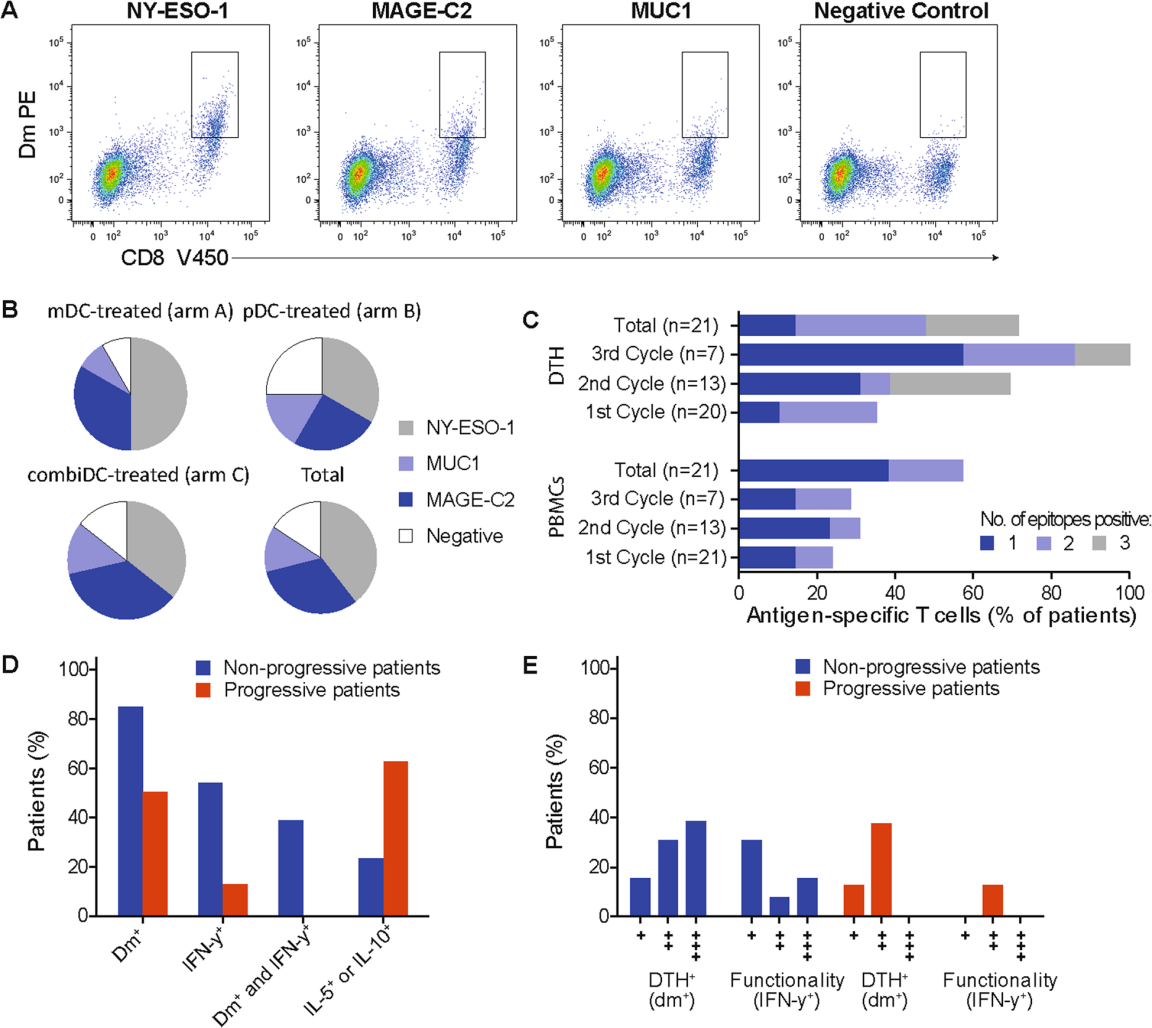
Immunological responses in the DTH skin-test and in blood. **a** Example of flow cytometric analysis of SKILs of patients combiDC-04. SKILs were stained with dextramers encompassing HLA-A0201-specific peptides of NY-ESO-1, MAGE-C2 and Mucin-1 (MUC1) or with a negative control (HLA-B*0801) and with anti-CD8. Tumor antigen-specific T cells were detected against all 3 tumor-associated antigens**. b** Tumor-associated antigen-specific responses in DTH skin-tests. NY-ESO-1-, MAGE-C2 and MUC1-specific T cell responses are presented per study arm and in total. **c** Number of antigen-specific responses in DTH skin-tests and in blood. Results are presented per vaccination cycle and in total. **d** Radiological non-progressive patients (*n* = 13) are defined as patients with the absence of disease progression within 6 months. Radiological progressive patients (*n* = 8) are defined as patients with progressive disease within 6 months. Presented are percentages of non-progressive and progressive patients with a positive DTH skin-test (tetramer/dextramer positive, dm^+^) for at least one epitope, IFN-y producing SKILs (IFN-y^+^), presence of both dm^+^ and IFN-y^+^ SKILs, and dominant IL-5^+^- or IL-10^+^-skewed immune responses, demonstrated by higher IL-5 or IL-10 production compared to IFN-y production in supernatant of antigen-challenged SKILs. **e** The presence of dm^+^ antigen-specific T cells and IFN-y-producing (IFN-y^+^) SKILs are shown for patients with non-progressive disease (*n* = 13) and progressive patients (*n* = 8). +: 1 epitope; ++: 2 epitopes; +++: 3 epitopes recognized. DTH: delayed-type hypersensitivity; dm: dextramer; PBMCs: peripheral blood mononuclear cells; PE: phyco erytrin; SKILs: skin-test infiltrating lymphocytes

DTH skin-tests were performed after each cycle of DC vaccinations to study NY-ESO-1-, MAGE-C2- and MUC1-specific T cell responses (Fig. 1a). NY-ESO-1-specific CD8^+^ T cells were detected in skin biopsies in 15 patients (71%). MAGE-C2- and MUC1-specific CD8^+^ T cells were found in 12 patients (57%) and 5 patients (24%), respectively. There were no significant differences in TAA-specific responses between patients vaccinated with mDCs, pDCs or combiDCs (Fig. 1b). In 15 of 21 patients (71%), tetramer- or dextramer-positive skin-derived T cells were observed for at least one TAA (Fig. 1c). In 7 of 20 patients (35%), these antigen-specific T cells were already detected after the first cycle of vaccinations. In 5 patients antigen-specific T cells were found against all 3 TAAs.

In peripheral blood prior to the start of DC vaccinations, in 7 of 21 patients (33%) NY-ESO-1-specific CD8^+^ T cells were detected. No MAGE-C2- or MUC1-specific T cells were found before DC vaccinations. Post-vaccination antigen-specific T cells could be detected in peripheral blood in 12 of 21 patients (57%). NY-ESO-1-, MAGE-C2- and MUC-1 specific T cells were detected in blood of 10 of 21 (48%), 4 of 21 (19%) and 2 of 21 (10%) patients, respectively. In 4 patients antigen-specific T cells in blood were found against more than one TAA (Fig. 1c).

SKILs were tested for their capacity to produce T helper 1 (Th1) cell cytokines (IFN-γ and IL-2) or T helper 2 (Th2) cell cytokines (IL-5 and IL-10) upon co-culture with the tumor antigen peptides. Th1-type cytokines are proinflammatory, whereas Th2-type cytokines have a suppressive action and dampen the immune responses. IFN-γ production (IFN-γ^+^) was detected in 8 of 21 patients (31%). In radiological non-progressive patients, both induced tumor antigen-specific T cells (tetramer/dextramer^+^ (dm^+^)) and functionality (IFN-γ^+^) was observed in 5 of 13 patients (38%) compared to 0 of 8 in radiological progressive patients (0%) (Fig. 1d). Recognition of multiple epitopes by induced antigen-specific T cells and IFN-γ^+^ was seen more frequently in patients with non-progressive disease (Fig. 1e). In 5 of 8 patients (63%) with radiological progression, we found a dominant IL-5- or IL-10-skewed immune response, compared to 3 of 13 patients (23%) with non-progressive disease (Fig. 1d).

#### Clinical outcome

**Fig. 2:**
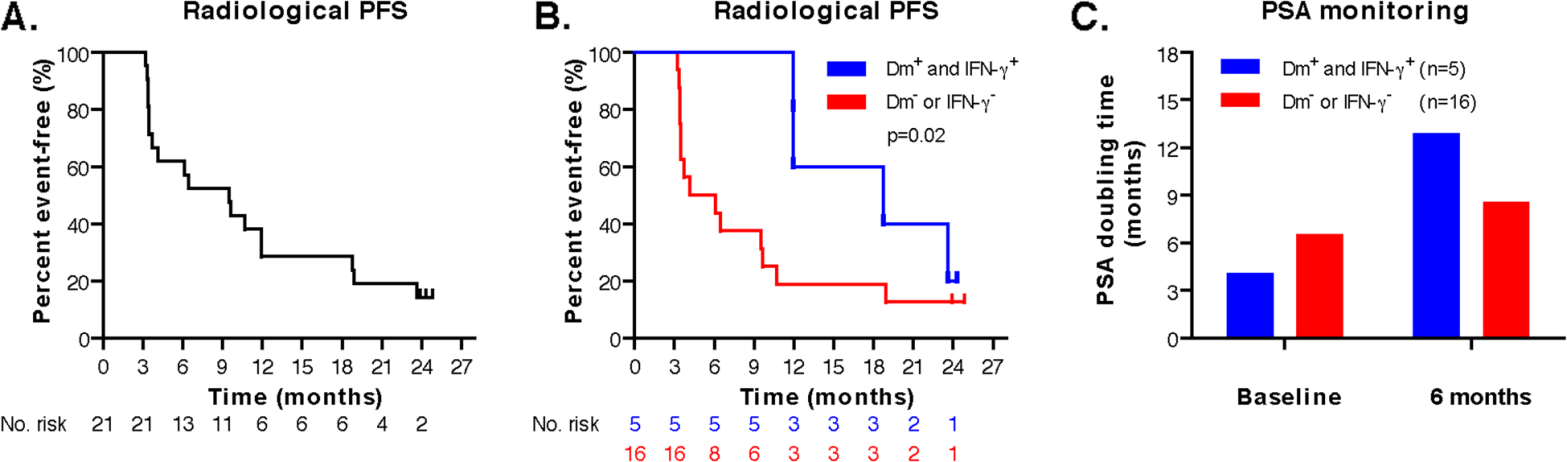
Radiological progression-free survival and biochemical responses. **a** Kaplan-Meier analysis of rPFS of all patient determined by a log-rank test. **b** Kaplan-Meier analysis of rPFS of patients with (dm^+^ and IFN-y^+^) or without (dm^−^ or IFN-y^−^) the presence of functional antigen-specific T cells in skin biopsies was determined by a log-rank test. **c** PSA doubling tome during DC vaccination therapy in dm^+^ and IFN-y^+^ patients (*n* = 5) and dm^−^ or IFN-y^−^ patients (*n* = 16)

**Fig. 3:**
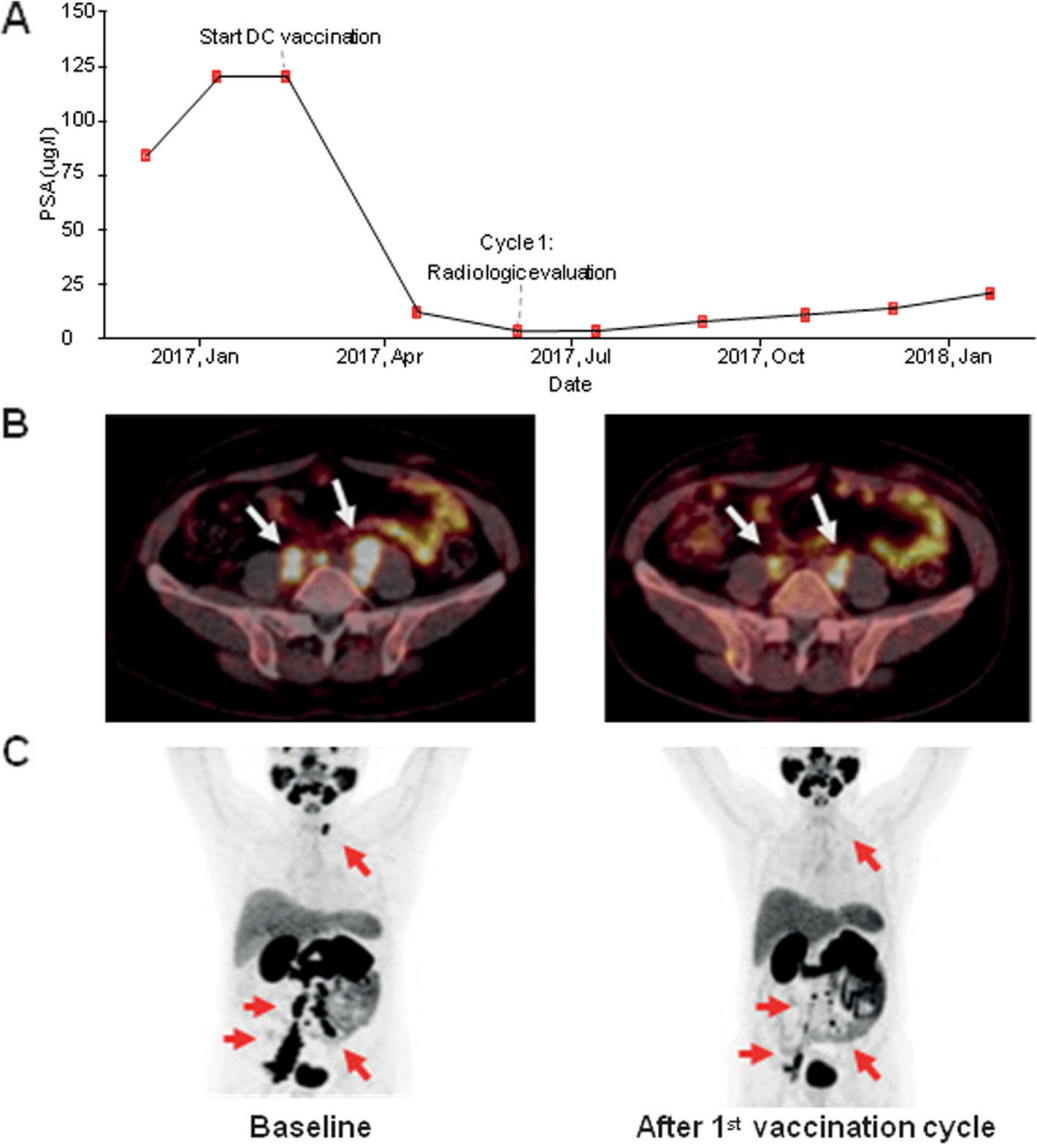
Biochemical and radiological response upon first DC vaccination cycle of patient combiDC-07. **a** Biochemical analysis shows a PSA normalization upon the first cycle of DC vaccinations. **b** Fused ^68^Ga-prostate-specific membrane antigen PET/CT images showed a significant reduction of bilateral para-iliac and para-aortic lymph node metastases, right inguinal node metastases and a left supraclavicular lymph node metastasis after the 1st cycle of DC vaccinations. Lymph nodes are indicated with white arrows. **c** Maximal intensity projection images. Lymph nodes are indicated with red arrows

Of the 21 included patients, in 1 patient (5%) a partial radiological response was observed. Stable disease that persisted > 6 months was seen in 12 patients (57%). In 8 patients (38%) disease progression was observed within 6 months. Median rPFS for all patients was 9.5 months (range: 3.2–24.8* months). The 6- and 12-months rPFS was 62% en 29%, respectively (Fig. 2a). There was no significant difference between the three treatment arms; in the mDC group the rPFS was 12.0 months (range 3.4–24.8* months), in the pDC group 10.7 months (range 3.4–23.9* months) and 4.2 months (range 3.2–12.0 months) in the combiDC group. The presence of functional antigen-specific T cells correlated with longer rPFS. In dm^+^ and IFN-γ^+^ patients (*n* = 5) median rPFS was found to be 18.8 months compared to 5.1 months in dm^−^ patients or patients without IFN-γ-producing antigen-specific T cells (*n* = 16; *p* = 0.02, Fig. 2b). Dm^+^ and IFN-γ^+^ patients showed longer PSAdt at 6 months compared to dm^−^ patients or patients without IFN-γ-producing antigen-specific T cells (mean PSAdt 12.9 months vs. 8.6 months, Fig. 2c). A decrease in PSA level was detected only in 2 of 21 patients. One of these patients (combiDC-07) showed a > 99% PSA-decrease which co-occurred with a partial radiological response (Fig. 3). Median OS was not reached. The median follow-up of all patients is 27.2 months (range 10.7–41.2* months). Reversed Kaplan Meier estimate of the median follow-up was not reached, to take censored casus into account. To date 8 patients deceased during the study period, 7 PCa-related deaths occurred and there was one non-PCa-related death due to a ruptured type A aortic dissection (Table 3). OS appeared longer in dm^+^ and IFN-γ^+^ patients (*n* = 5) versus dm^−^ patients/patients without IFN-γ-producing antigen-specific T cells (*n* = 16) (Additional file 7: Figure S7). 5 patients (3 mDC, 1 pDC and 1 combiDC treated) had a skeletal-related event (SRE). The median time to SRE was not reached (range 3.6–21.8 months after apheresis). These 5 patients had bone pain secondary to bone metastases and were treated with palliative radiotherapy. Four of them were post radiotherapy treated with docetaxel-based chemotherapy. In 7 patients, docetaxel was initiated (range 3.7–29.2 months after apheresis) (Additional file 6: Figure S6). Median time to opiate use for cancer-related pain and median time to ECOG performance score deterioration was also not reached. Seven patients started with opioids (range 1.4–20.1 months after apheresis). Ten patients had a decline in ECOG performance score (3 mDC, 3 pDC and 4 combiDC treated; range 1.5–20.1 months after apheresis). Details of clinical, immunological, immunohistochemical and sequencing outcomes are presented in Table 3, Additional file 6: Figure S6, Additional file 7: Figure S7 and Additional file 8: Table S1.

**Table 3:** Clinical and immunological outcome

Blood-derived DC treated patient^~^	Measurable disease sites at baseline^a^	Radiological progression-free survival (mo)^b^	Overall survival (mo)	Dm^c^ SKILs^e^	IFN-γ^c^ SKILs^e^	Number of vaccinations
mDC-01	Bone	23.6	36.1^c^	+	+	9
mDC-02	–	24.3^c^	34.8^c^	+	+	9
mDC-03	–	24.8^c^	34.8^c^	+	–	9
mDC-04	LN, bone	3.4	28.0	+	–	3
mDC-05	LN	12.0	30.2^c^	+	+	6
mDC-06	Bone	3.4	23.3	–	+	3
mDC-07	LN, bone	3.4	17.1	+	–	3
pDC-01	LN	18.8	36.8^c^	+	+	9
pDC-02	Bone	6.4	24.9	–	+	6
pDC-03	LN, bone	3.4	20.0	–	–	3
pDC-04	LN	18.9	27.6^c^	+	–	9
pDC-05	Bone	6.1	27.6^c^	–	+	6
pDC-06	LN	23.9^c^	37.8^c^	+	–	9
pDC-07	LN, bone	10.7^d^	10.7	+	–	6
combiDC-01	Bone	4.2	41.2^c^	–	–	3
combiDC-02	Bone	3.2	21.7	+	–	3
combiDC-03	–	3.7	20.4	+	–	3
combiDC-04	Bone	9.5	27.2^c^	+	–	6
combiDC-05	Bone	3.4	26.7^c^	–	–	3
combiDC-06	LN, bone	9.7	25.6^c^	+	–	6
combiDC-07	LN, bone	12.0	24.4^c^	+	+	9

*DC* dendritic cells, *Dm* dextramer, *LN* lymph nodes, *mo* months, *SKILs* skin-infiltrating lymphocytes

^a^measurable disease sites were determined on advanced imaging with contrast-enhanced ^68^Ga-prostate-specific membrane antigen (PSMA) PET/CT scans, for RECIST 1.1 and ferumoxtran-10-enhanced MRIs and MRI bones for PCWG2 criteria

^b^radiological responses were assessed on contrast-enhanced ^68^Ga-PSMA PET/CT scans, ferumoxtran-10-enhanced MRIs and MRI lymph nodes for RECIST 1.1 and MRI bones for PCWG2 criteria. In case of progressive disease according to RECIST 1.1 and/or PCWG2 criteria a confirmatory MRI lymph nodes and bones was performed 6–8 weeks later. The date used for calculation of progression-free survival was the first date at which progression criteria were met (the date of unconfirmed progression of disease)

^c^progression-free survival or overall survival endpoint not reached in this patient

^d^patient had stable disease according to RECIST 1.1 and PCWG2 criteria. At 10.7 months after apheresis patient deceased due to a ruptured type A acute aortic dissection

^e^tetramer- or dextramer-positivity (dm^+^) or IFN-γ-positivity of SKILs if at least for one epitope CD8^+^ dm^+^ or IFN-γ^+^ T cells were detected

#### Clinical outcome related to immunohistochemical results

**Fig. 4:**
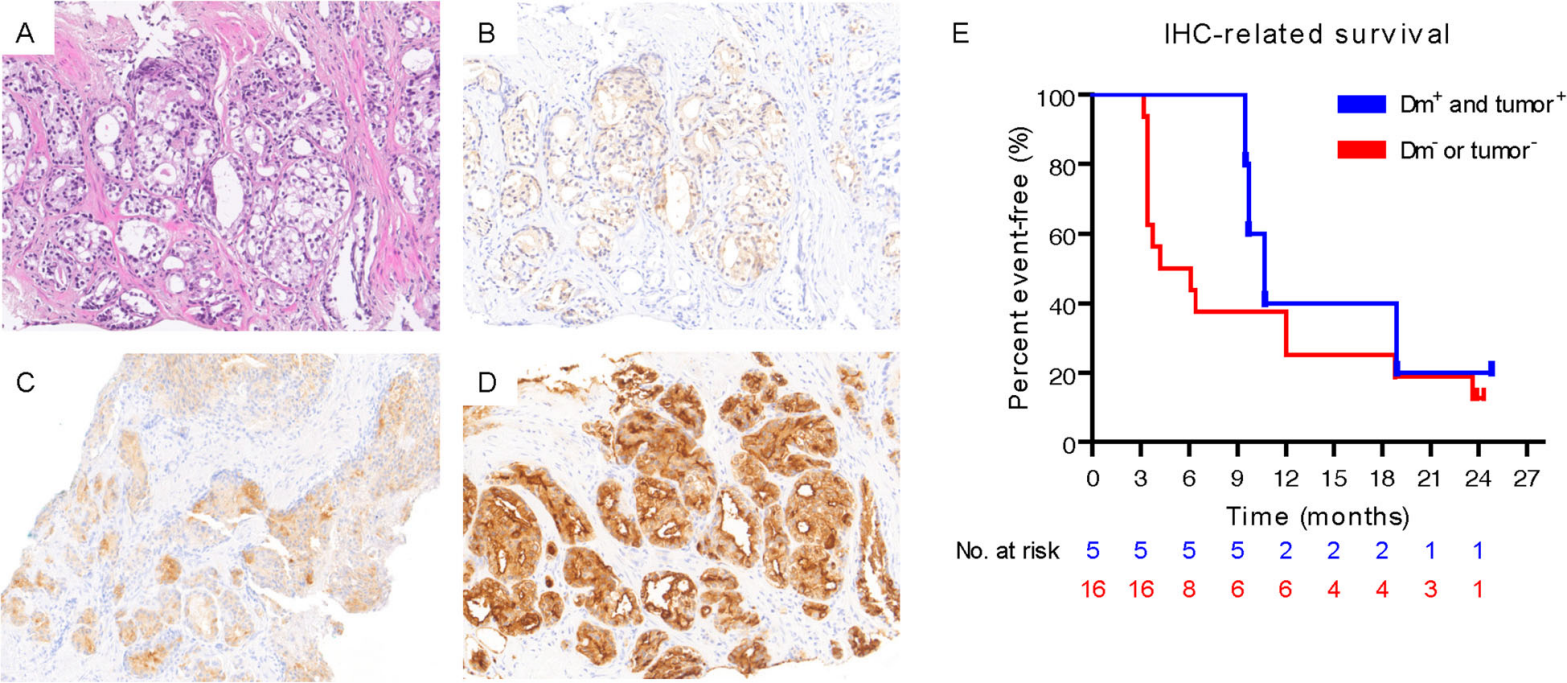
Expression of NY-ESO-1, MAGE-C2 and MUC1 and its relation to antigen-specific T cells in skin biopsies. **a**-**d** Representative immunohistochemical images showing (**a**) haematoxylin and eosin stain (H&E stain) and the expression of (**b**) NY-ESO-1, (**c**) MAGE-C2 and (**d**) MUC1. **e** Kaplan-Meier curve of rPFS in patients with or without the presence of antigen-specific T cells (dm^+^) in skin biopsies and expression of the same tumor-associated antigen in the tumor (dm^+^ and tumor^+^)

To study the effect of DC vaccination on TAA expression by the primary tumor, TAA expression was assessed on available prostate biopsies or radical prostatectomy tissue (Fig. 4a-d and Additional file 7: Table S1). Patients with TAA-specific T cells whose tumor expressed the same TAA (dm^+^ and tumor^+^; *n* = 5) had a median rPFS of 10.7 months (range: 9.5–24.8*). Patients that did not have matching TAA-specific T cells and TAA-expression of the tumor (dm^+/−^ and tumor^−^; *n* = 16), had a median rPFS of 5.2 months (range: 3.2–24.3*) (Fig. 4b). This difference was not statistically significant. In two patients who progressed after DC vaccination, loss of MUC1 expression by the tumor was observed. In one of these patients, MUC-1-specific T cells were detected. Tumor PD-L1 expression was studied in 10 patients. In two of these patients tumor PD-L1 expression post-vaccination was ≥1%. One of them was a dm^+^ and IFN-γ^+^ patient showing tumor PD-L1 expression of 60%. Tumors of all biopsied patients were microsatellite stable (Additional file 7: Table S1).

## Discussion

Patients with CRPC were vaccinated with DCs isolated directly from blood with a fully closed semi-automated system. Patients received mature mDCs (cDC2) and/or pDCs in order to induce tumor antigen-specific immune responses. We showed that vaccination with blood-derived DCs is safe and leads to the induction of antigen-specific T cells in the majority of patients. The induction of both antigen specific and functional T cells correlates with beneficial clinical outcome. In these small cohorts, no significant differences between DC subsets were observed, although responses to mDCs might be most promising (Additional File 6: Figure S6). Clinical efficacy of single DC subset vaccination or the combination of mDCs and pDCs will be assessed further in follow-up phase II/III studies.

DTH-skin test-derived and IFN-γ-producing antigen-specific T cells were detected more frequently in SKIL cultures of patients with non-progressive disease compared to those with progressive disease. Thus, the presence of functional antigen-specific T cells might be indicative for a clinical beneficial response to DC vaccination. This is in line with our previous study in stage IV melanoma patients vaccinated with CD1c^+^ DCs (cDC2), in whom the presence of functional tumor antigen-specific T cells in SKIL cultures coincided with improved clinical outcome [24]. Despite the fact that our study is not designed for clinical outcome assessment, we found a difference in median rPFS between patients with functional antigen-specific T cells (18.8 months; *n* = 5) and patients with no functional antigen-specific T cells (5.1 months; *n* = 16). In addition, observed survival times of the patients might potentially indicate a difference in OS in favor of dm^+^ and IFN-γ^+^ patients. However, this finding has to be interpreted with caution. Firstly, the limited size renders our trial underpowered concerning reliable statements on the OS. Secondly, the number and type of subsequent therapies likely influenced the OS of these patients, clouding the direct effects of DC vaccination hereon (Additional File 6: Figure S6).

Following DC vaccination the rPFS of patients with functional antigen-specific T cells appeared comparable to median rPFS reported for abiraterone- (16.5 months) and enzalutamide-treated (20.0 months) men with metastatic CRPC who were chemotherapy naive [47, 48]. It is important to note that there is a considerable risk for guarantee-time bias [49] when correlating immunological responses of multiple vaccination cycles to clinical responses since patients who completed more than one vaccination cycle had a higher chance of developing IFN-γ-producing antigen-specific SKILs [50]. The clinical impact of DC vaccination and validation of an immunological response readout as a surrogate endpoint will have to be studied in a larger phase II or III clinical trial.

In contrast to conventional response assessment using contrast enhanced-CT scans and radioisotope bone scans, we used ^68^Ga-PSMA PET/CT scans [33], including thin-section diagnostic CT (3 mm) and ferumoxtran-10-enhanced MRIs [34, 35] for disease evaluation according to RECIST version 1.1 [36] and PCWG2 criteria [37, 51]. To assess immune unconfirmed progressive disease immune-related response criteria and the iRECIST criteria were used [38–41]. We introduced both contrast-enhanced ^68^Ga-PSMA PET/CT scans and ferumoxtran-10-enhanced MRIs to be able to study disease distribution, disease biology and host reaction within the tumor microenvironment of both measurable as non-measurable lesions [52]. Therefore, we have decided to use the best imaging modalities since it is very likely that these imaging modalities will become standard of care in the next five years. The functional imaging data will be reported elsewhere (manuscript in preparation).

Vaccination with blood-derived DCs resulted in only low-grade toxicity, that was similar to our previous studies [23, 24]. There were four patients who experienced possible vaccine-related symptoms of a grade 2 upper respiratory tract infection. These patients clinically recovered after treatment with oral antibiotics. Therefore, in our opinion, these were not related in retrospect, but this adverse event has to be monitored during ongoing and future trials with DCs.

The relation between the presence of antigen-specific T cells and tumor-antigen expression on PCa tissue was not obvious. PCa biopsies were obtained years before patients developed CRPC. From literature is known that in localized PCa the expression of MAGE-C2 is significantly lower (3%), compared to the CRPC setting (23%) [53]. The same accounts for NY-ESO-1 expression, which is positive in 3% of patients with localized PCa and 15% of patients with CRPC [54]. Also, an association of MUC1 upregulation with the development of CRPC is previously reported [55]. Retrospectively, compulsory tumor biopsies taken in the CRPC setting, prior to start of DC vaccination, would have been most informative for assessing associations between antigen expression and induction of antigen-specific T cells. In follow-up trials, tumor antigen-expression in fresh biopsies will be included, which may serve as either a selection criterion, or as an exploratory endpoint.

Our study is the second trial worldwide investigating immune responses upon vaccination with blood-derived DCs in advanced PCa. Previously, Prue and colleagues performed a phase I trial with HLA-A*0201 peptide-loaded CD1c^+^ DCs in 12 prostate cancer patients [56]. This vaccine was also very well tolerated, showing only grade 1–2 adverse events. In contrast to our study, in none of the patients in the study of Prue et al. tumor antigen-specific immune responses were observed and only 25% of patients developed a DTH skin-test response to the control antigens after vaccination. This might be due to the difference in administration route. We vaccinated patients intranodally, Prue and colleagues vaccinated their patients intradermally and intravenously. Due to the low numbers of DCs available, intradermally and intravenously administered blood-derived DCs might not have reached the lymph nodes in sufficient numbers. Although only low numbers of DCs are necessary to induce an immune response [57], direct intranodal injection of these scarce DCs might be more effective for T cell priming.

The therapeutic landscape for patients with CRPC is changing drastically with the vast number of potential single agent therapies and combination therapies that have been approved and are under investigation for CRPC. Until now, the clinical outcome of immune checkpoint inhibitors is disappointing in advanced PCa [14, 15]. However, the PD-1 inhibitor pembrolizumab shows antitumor activity in patients with evidence of progression on enzalutamide [58] and in the docetaxel-refractory setting (Keynote-199 trial; NCT02787005). Several trials with immune checkpoint inhibitors as a single agent treatment or as combination therapy are currently ongoing in both unselected as in immunogenic subtypes, such as those harboring microsatellite instability, high tumor mutational load or biallelic inactivation of CDK12 or BRCA2 [59].

Cellular immunotherapy with sipuleucel-T showed OS benefit in the phase III IMPACT trial [9]. On the contrary, the GM-CSF secreting GVAX cell line did not improve clinical outcome, but had similar survival data when compared to docetaxel in a phase III clinical trial [60, 61]. Since the study was designed as a superiority trial no statement could be made that these treatments were equally effective. The phase III trial of pox-virus-based co-stimulatory molecule-assisted vaccine PROSTVAC +/− granulocyte-macrophage colony-stimulating factor was stopped early. It had no effect on OS [62]. The VIABLE trial (docetaxel +/− DC vaccination, NCT02111577) is currently undergoing phase III evaluation.

Our DC vaccination strategy aims at inducing cytotoxic T lymphocytes. However, immune cell recruitment to the tumor and efficient tumor cell killing by cytotoxic T cells is probably less effective in patients with CRPC compared to patients with localized cancer. Indeed, recent studies show that in advanced cancer patients, the immunosuppressive state of the tumor, caused by regulatory T cells, myeloid-derived suppressor cells, expression of PD-L1 and production of immunosuppressive cytokines hampers the immune response towards the tumor [63–66]. Induced T cell responses frequently fail to fully eliminate cancer, because of an exhausted or dysfunctional state of the T cells [67]. This can be caused by an imbalance between T cell invigoration and reinvigoration and tumor burden [64]. We showed promising preliminary clinical outcome for patients with functional antigen-specific T cells. However, most single agent immunotherapies will fail to completely eliminate cancer cells in the majority of advanced cancer patients. Future trials could focus on combination therapies, such as a cellular-based immunotherapy and immune checkpoint inhibition. Another strategy is treating early-stage PCa in order to precede cancer-induced immunosuppressive mechanisms.

This trial was not designed to study the effects of sequential follow-up therapies for patients with CRPC. Most frequent subsequent treatments were abiraterone acetate, enzalutamide and docetaxel (Additional file 6: Figure S6). Current data showed no hampering of the therapeutic effect of these agents. In addition, it is hypothesized that immunotherapy has a delayed effect on the tumor growth curve evoking durable and adaptable anti-cancer immune responses over an extended time period [68]. However, it remains a major challenge how to position the different therapies in the current treatment strategy of patients with CRPC. There are still unanswered questions regarding the preferred therapy approach (sequence or combination therapy), the timing of therapies and the relative efficacy of every single treatment. However, harboring vaccination-induced functional antigen-specific T cells might be beneficial even after disease progression upon DC vaccination. This relative efficacy might have clinical benefit during subsequent therapies .

In conclusion, we demonstrated that vaccination with blood-derived mDCs (cDC2) and/or pDCs induced functional tumor antigen-specific immune responses in patients with CRPC. Patients harboring functional antigen-specific T cells showed a significantly increased median rPFS and might have an OS benefit compared to patients without these cells. This immune correlate might be indicative for a beneficial response to DC vaccination and opens up new opportunities for future immunotherapy trials with the intention of long-term cancer control.

## Supplementary information

**Additional file 1: Figure S1.** Flowchart for patient accrual and treatment with DC vaccinations. * In two patients randomized for treatment with combiDCs the final mDC product did not fulfill the release criteria. Therefore, the patients were vaccinated with pDCs only. Because the primary endpoint of the study was immunological, two extra patients were randomized within the combiDC arm.

**Additional file 2: Figure S2.** Schematic representation of (A) the treatment schedule and B) dendritic cell isolation and culture.

**Additional file 3: Figure S3.** Myeloid and plasmacytoid vaccine characteristics. (A) Purity of freshly isolated pDC and mDC was analyzed by flow cytometry and based on expression of CD123 and BDCA2 (pDC) or CD1c with absence of CD20 (mDC). (B) Yield of pDCs and mDCs after isolation with CliniMACS Prodigy. (C) Phenotype, (D) viability and (E) cytokine production of pDC and mDC after maturation with protamine-mRNA complexes. Phenotype was analyzed by flow cytometry. Cytokine production was analyzed in the supernatant by cytometric bead array.

**Additional file 4: Figure S4.** Positive controls for antibody validation. Validation of NY-ESO-1, MAGE-C2 and MUC1 antibodies for immunohistochemistry in positive control tissue (testicular or tonsil tissue).

**Additional file 5: Figure S5.** KLH-specific immune responses before and after DC vaccination. (A) KLH-specific T cell proliferation was analyzed before the first vaccination and after DC vaccination. Proliferative response to KLH is given as proliferation index (proliferation with KLH/proliferation without KLH) and the maximal index during DC vaccination therapy is shown for each patient. Results are presented per study-arm. A paired t-test was used to compare responses before and after vaccination. (B) KLH-specific IgG antibodies were quantitatively measured after each vaccination cycle in sera of vaccinated patients. Humoral responses upon DC vaccination shown per arm. Maximum total IgG titers during DC vaccination therapy are presented for each patient. Each dot represents one patient. A paired t-test was used to compare responses before and after vaccination. * *p* = < 0.05.

**Additional file 6: Figure S6.** Patient response, survival and systemic treatments since start of DC vaccination. Swimmer plot showing long-term clinical course for each patient. An arrow indicates that the patient is alive at last follow-up. A cross indicates patient demise.

**Additional file 7: Figure S7.** Kaplan-Meier estimates of survival. Kaplan-Meier analysis of overall survival of patients with (dm^+^ and IFN-y^+^) or without (dm^−^ or IFN-y^−^) the presence of functional antigen-specific T cells in skin biopsies.

**Additional file 8: Table S1.** Immunological, immunohistochemical and whole genome sequencing data.

## Data Availability

The data that support the findings of this clinical trial are available from the authors upon request.

## Abbreviations

combiDCs: combined CD1c^+^ myeloid and plasmacytoid dendritic cells
CRPC: Castration-resistant prostate cancer
CTCAE: Common Terminology Criteria for Adverse Events
DCs: Dendritic cells
dm^+^: tetramer or dextramer-positive
DTH: Delayed-type hypersensitivity
ECOG: Eastern Cooperative Oncology Group
GMP: Good Manufacturing Practices
IFN-γ^+^: IFN-γ-producing
KLH: Keyhole limpet hemocyan
mDCs: CD1c^+^ myeloid dendritic cells/cDC2
OS: Overall survival
PBMCs: Peripheral blood mononuclear cells
PCa: Prostate cancer
PCWG2: Prostate Cancer Clinical Trials Working Group 2
pDCs: plasmacytoid dendritic cells
PSA: Prostate-specific antigen
PSAdt: Prostate-specific antigen doubling time
PSMA: Prostate-specific membrane antigen
RECIST: Response Evaluation Criteria In Solid Tumours
rPFS: radiological progression-free survival
SKILs: Skin-test infiltrating lymphocytes
TAA: Tumor-associated antigen
Th1: T helper 1; Th2: T helper 2
