# Supplementary figures and images for: Blood-derived dendritic cell vaccinations induce immune responses that correlate with clinical outcome in patients with chemo-naive castration-resistant prostate cancer
Source: J Immunother Cancer. 2019 Nov 14;7:302. doi: 10.1186/s40425-019-0787-6 (PMC6854814; doi:10.1186/s40425-019-0787-6)

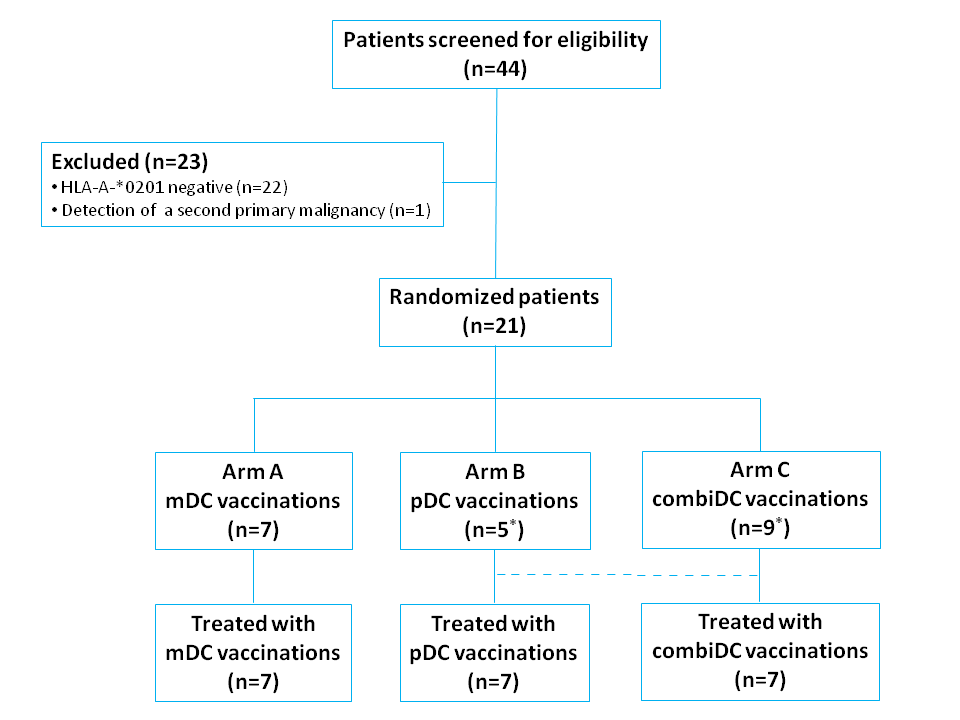

Supplement: Supplementary file 1 — Additional file 1: Figure S1. Flowchart for patient accrual and treatment with DC vaccinations. * In two patients randomized for treatment with combiDCs the final mDC product did not fulfill the release criteria. Therefore, the patients were vaccinated with pDCs only. Because the primary endpoint of the study was immunological, two extra patients were randomized within the combiDC arm. [file 40425_2019_787_MOESM1_ESM.tif]

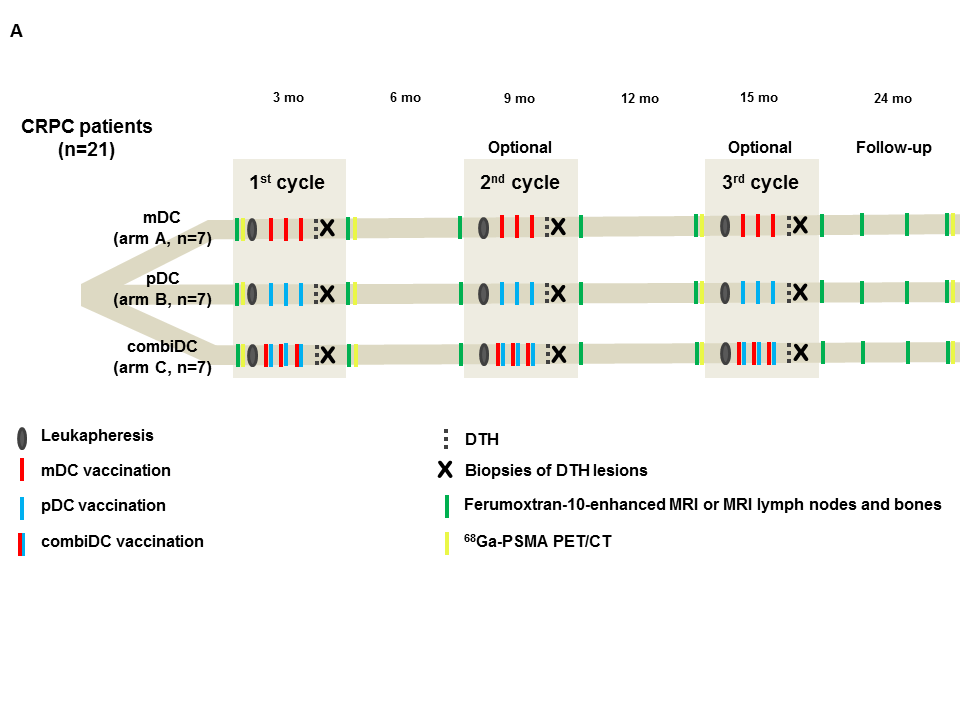

Supplement: Supplementary file 2 — Additional file 2: Figure S2. Schematic representation of (A) the treatment schedule and B) dendritic cell isolation and culture. [file 40425_2019_787_MOESM2_ESM.zip › Figure S2A.tif]

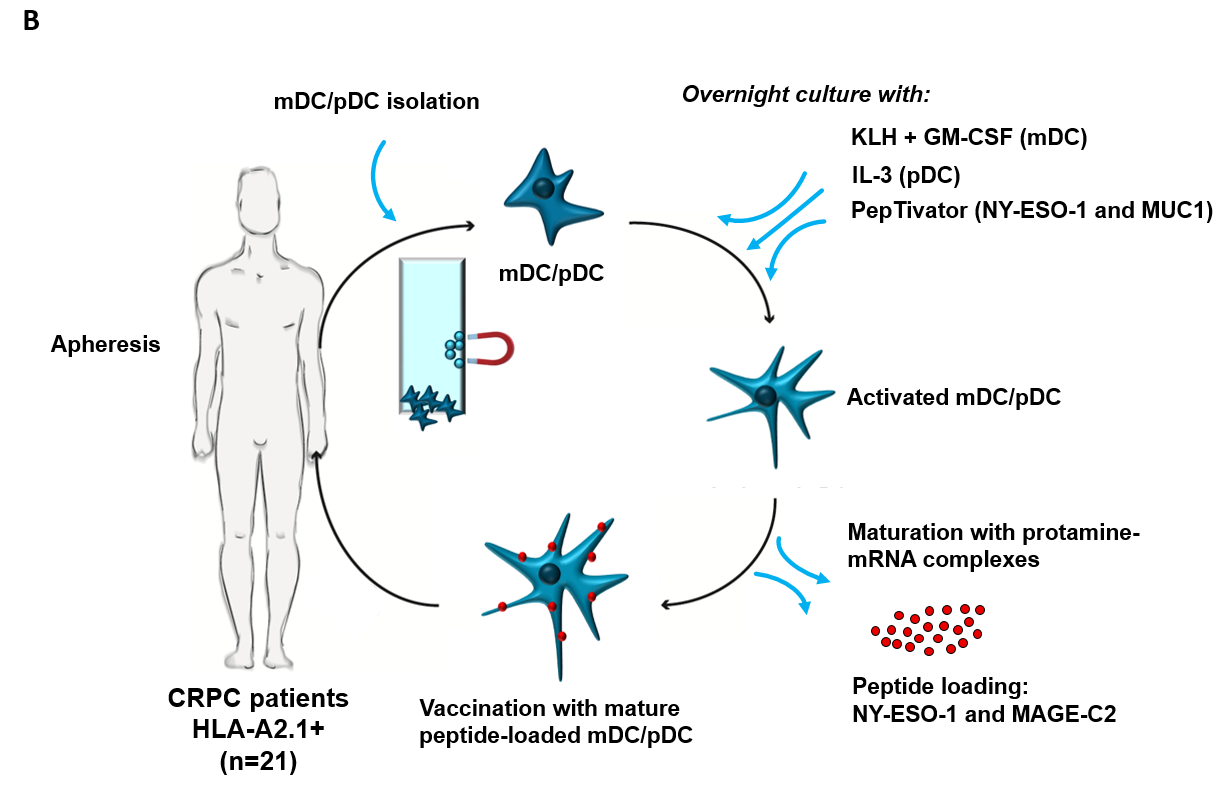

Supplement: Supplementary file 2 — Additional file 2: Figure S2. Schematic representation of (A) the treatment schedule and B) dendritic cell isolation and culture. [file 40425_2019_787_MOESM2_ESM.zip › Figure S2B.tif]

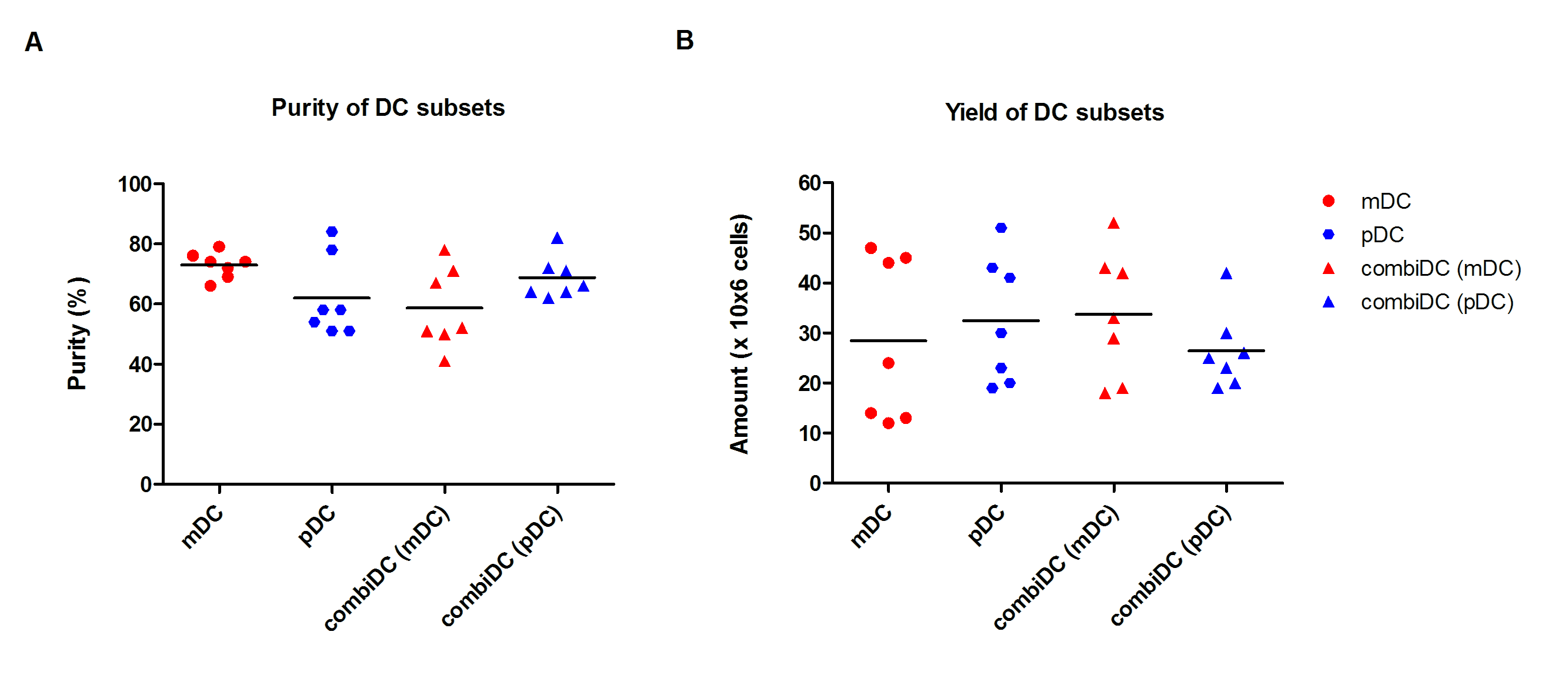

Supplement: Supplementary file 3 — Additional file 3: Figure S3. Myeloid and plasmacytoid vaccine characteristics. (A) Purity of freshly isolated pDC and mDC was analyzed by flow cytometry and based on expression of CD123 and BDCA2 (pDC) or CD1c with absence of CD20 (mDC). (B) Yield of pDCs and mDCs after isolation with CliniMACS Prodigy. (C) Phenotype, (D) viability and (E) cytokine production of pDC and mDC after maturation with protamine-mRNA complexes. Phenotype was analyzed by flow cytometry. Cytokine production was analyzed in the supernatant by cytometric bead array. [file 40425_2019_787_MOESM3_ESM.zip › Figure S3A and S3B.tif]

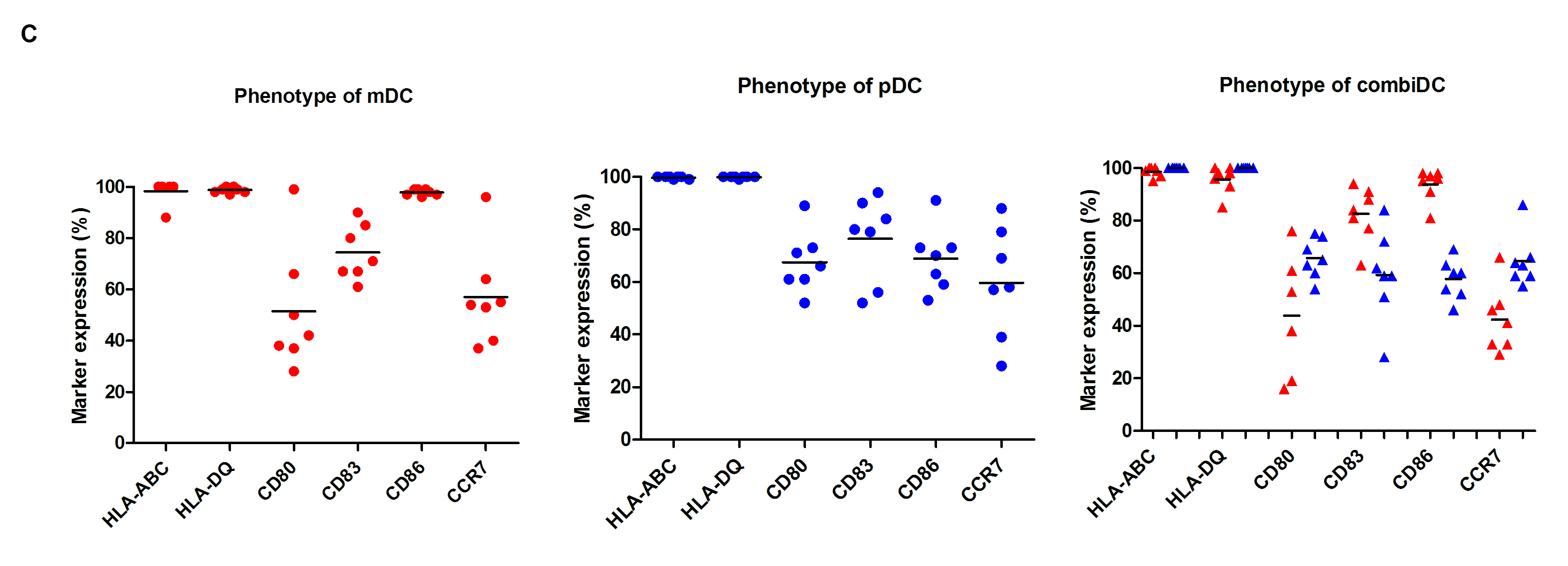

Supplement: Supplementary file 3 — Additional file 3: Figure S3. Myeloid and plasmacytoid vaccine characteristics. (A) Purity of freshly isolated pDC and mDC was analyzed by flow cytometry and based on expression of CD123 and BDCA2 (pDC) or CD1c with absence of CD20 (mDC). (B) Yield of pDCs and mDCs after isolation with CliniMACS Prodigy. (C) Phenotype, (D) viability and (E) cytokine production of pDC and mDC after maturation with protamine-mRNA complexes. Phenotype was analyzed by flow cytometry. Cytokine production was analyzed in the supernatant by cytometric bead array. [file 40425_2019_787_MOESM3_ESM.zip › Figure S3C.tif]

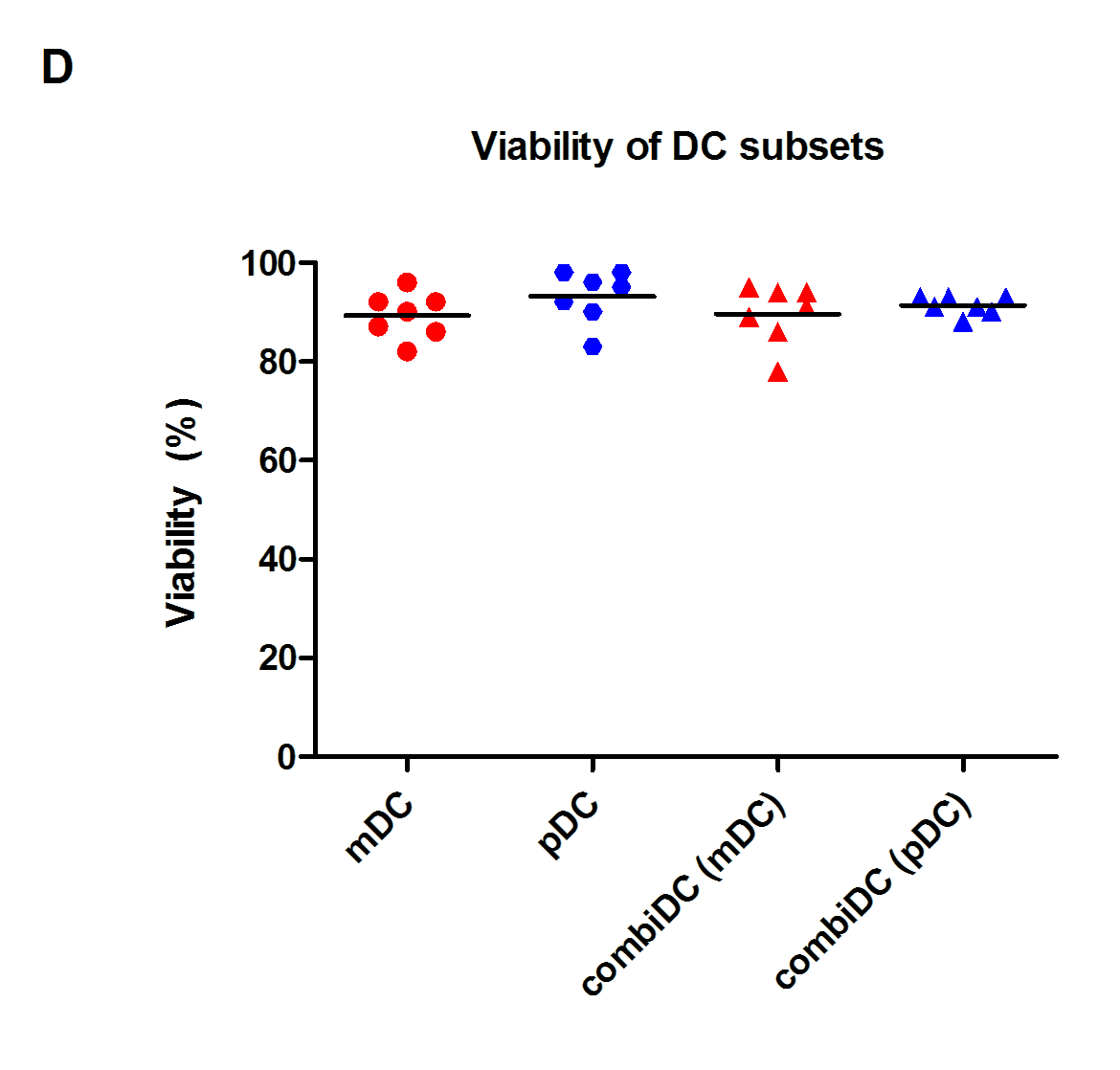

Supplement: Supplementary file 3 — Additional file 3: Figure S3. Myeloid and plasmacytoid vaccine characteristics. (A) Purity of freshly isolated pDC and mDC was analyzed by flow cytometry and based on expression of CD123 and BDCA2 (pDC) or CD1c with absence of CD20 (mDC). (B) Yield of pDCs and mDCs after isolation with CliniMACS Prodigy. (C) Phenotype, (D) viability and (E) cytokine production of pDC and mDC after maturation with protamine-mRNA complexes. Phenotype was analyzed by flow cytometry. Cytokine production was analyzed in the supernatant by cytometric bead array. [file 40425_2019_787_MOESM3_ESM.zip › Figure S3D.tif]

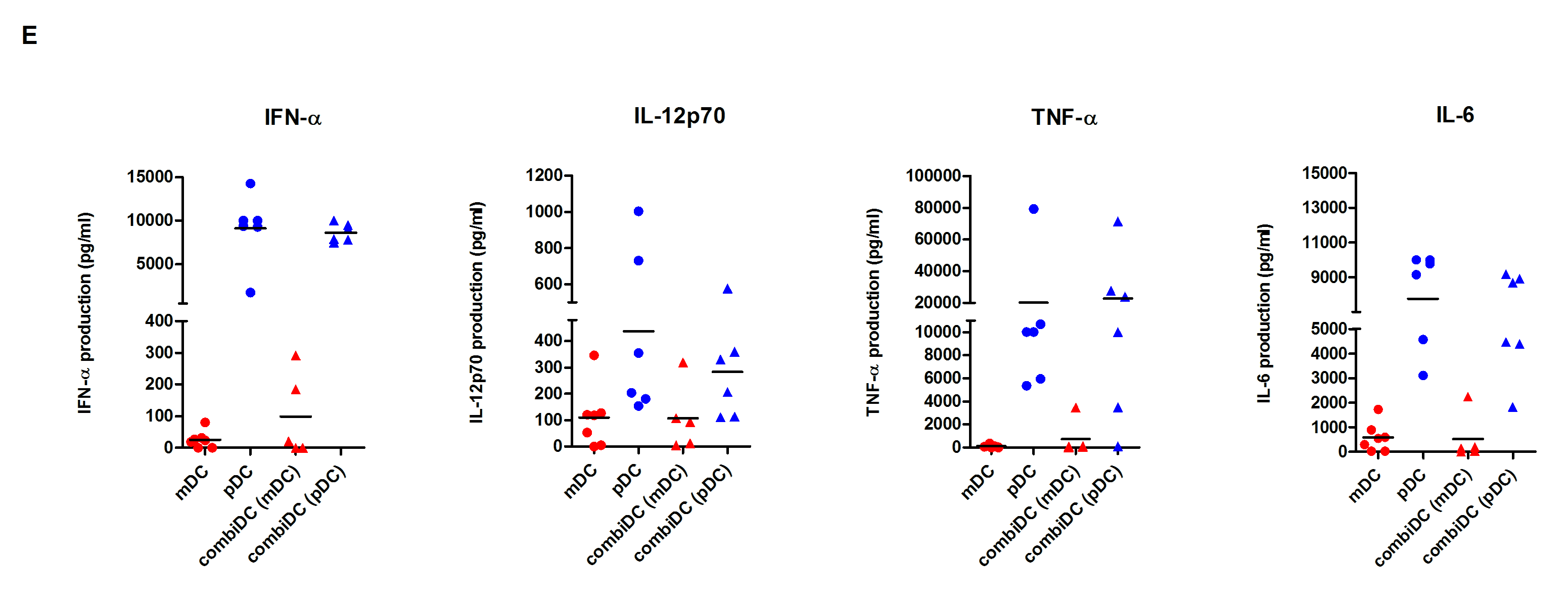

Supplement: Supplementary file 3 — Additional file 3: Figure S3. Myeloid and plasmacytoid vaccine characteristics. (A) Purity of freshly isolated pDC and mDC was analyzed by flow cytometry and based on expression of CD123 and BDCA2 (pDC) or CD1c with absence of CD20 (mDC). (B) Yield of pDCs and mDCs after isolation with CliniMACS Prodigy. (C) Phenotype, (D) viability and (E) cytokine production of pDC and mDC after maturation with protamine-mRNA complexes. Phenotype was analyzed by flow cytometry. Cytokine production was analyzed in the supernatant by cytometric bead array. [file 40425_2019_787_MOESM3_ESM.zip › Figure S3E.tif]

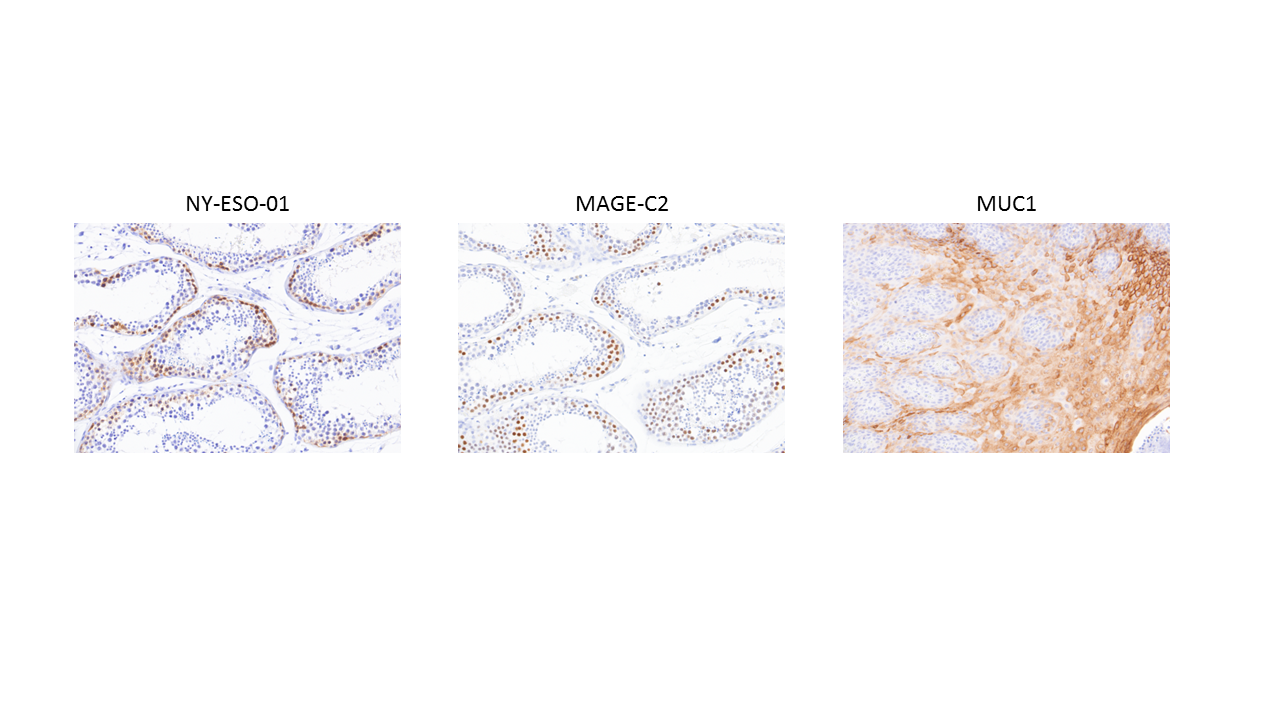

Supplement: Supplementary file 4 — Additional file 4: Figure S4. Positive controls for antibody validation. Validation of NY-ESO-1, MAGE-C2 and MUC1 antibodies for immunohistochemistry in positive control tissue (testicular or tonsil tissue). [file 40425_2019_787_MOESM4_ESM.tif]

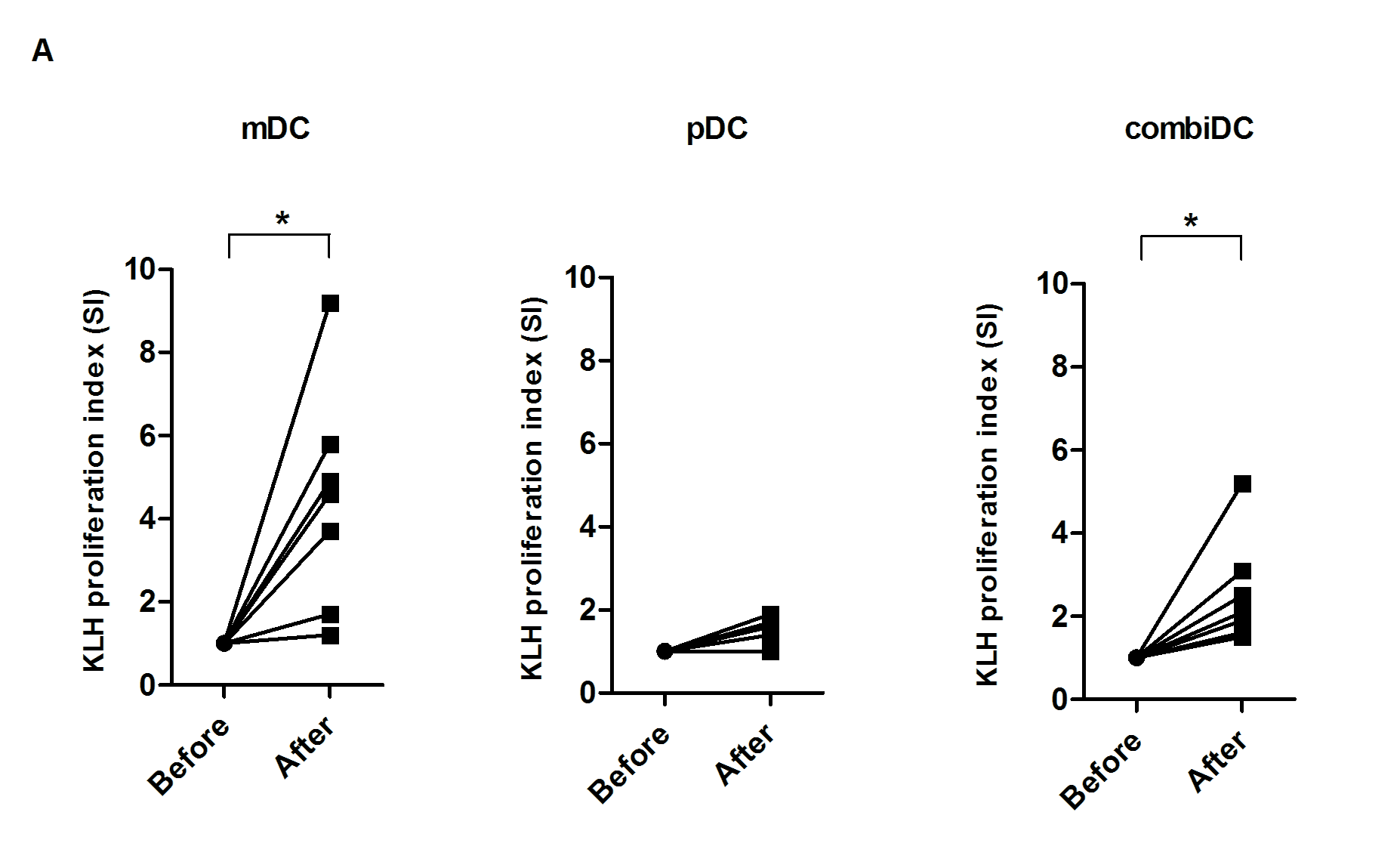

Supplement: Supplementary file 5 — Additional file 5: Figure S5. KLH-specific immune responses before and after DC vaccination. (A) KLH-specific T cell proliferation was analyzed before the first vaccination and after DC vaccination. Proliferative response to KLH is given as proliferation index (proliferation with KLH/proliferation without KLH) and the maximal index during DC vaccination therapy is shown for each patient. Results are presented per study-arm. A paired t-test was used to compare responses before and after vaccination. (B) KLH-specific IgG antibodies were quantitatively measured after each vaccination cycle in sera of vaccinated patients. Humoral responses upon DC vaccination shown per arm. Maximum total IgG titers during DC vaccination therapy are presented for each patient. Each dot represents one patient. A paired t-test was used to compare responses before and after vaccination. * p = < 0.05. [file 40425_2019_787_MOESM5_ESM.zip › Figure S5A.tif]

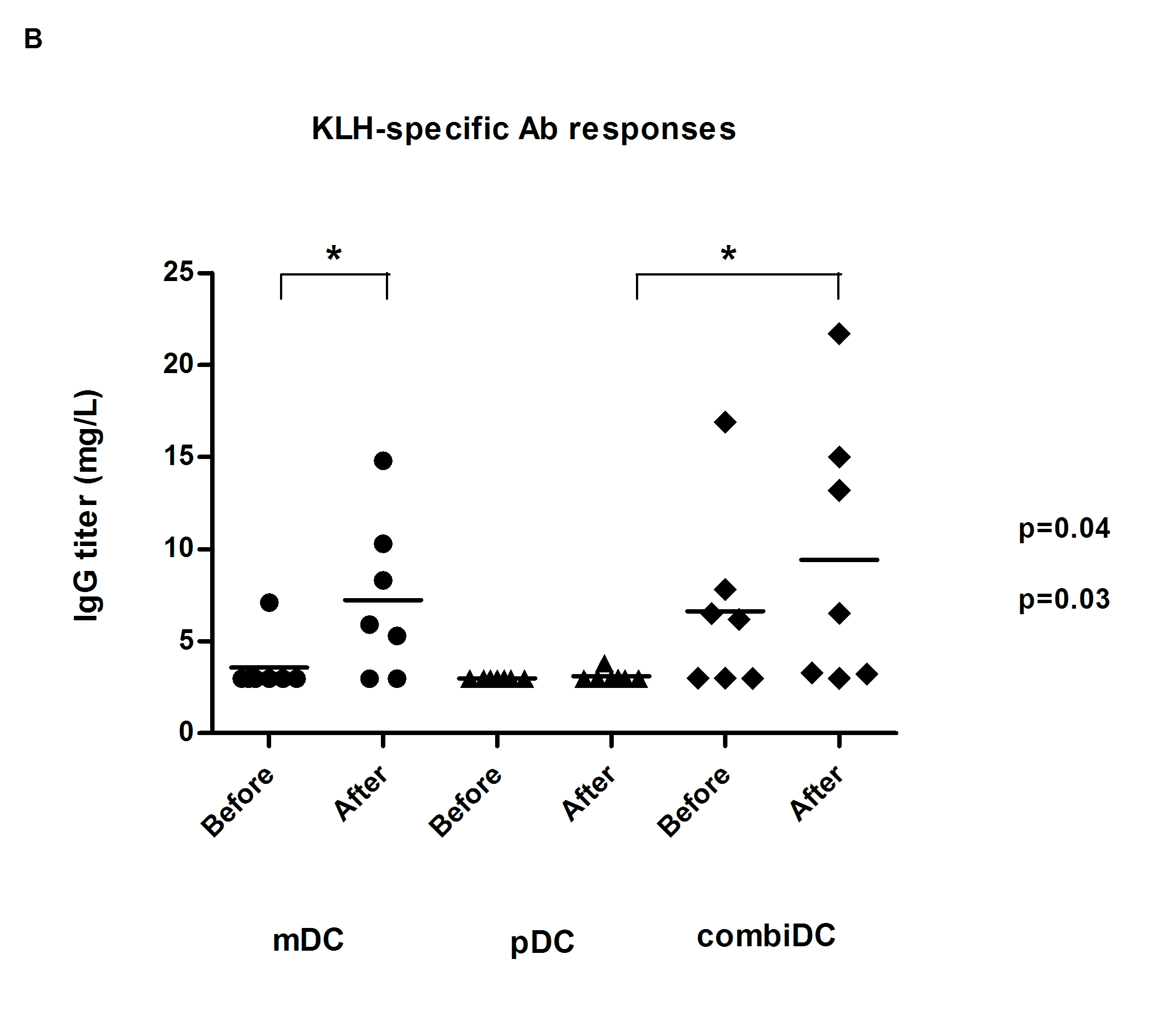

Supplement: Supplementary file 5 — Additional file 5: Figure S5. KLH-specific immune responses before and after DC vaccination. (A) KLH-specific T cell proliferation was analyzed before the first vaccination and after DC vaccination. Proliferative response to KLH is given as proliferation index (proliferation with KLH/proliferation without KLH) and the maximal index during DC vaccination therapy is shown for each patient. Results are presented per study-arm. A paired t-test was used to compare responses before and after vaccination. (B) KLH-specific IgG antibodies were quantitatively measured after each vaccination cycle in sera of vaccinated patients. Humoral responses upon DC vaccination shown per arm. Maximum total IgG titers during DC vaccination therapy are presented for each patient. Each dot represents one patient. A paired t-test was used to compare responses before and after vaccination. * p = < 0.05. [file 40425_2019_787_MOESM5_ESM.zip › Figure S5B.tif]

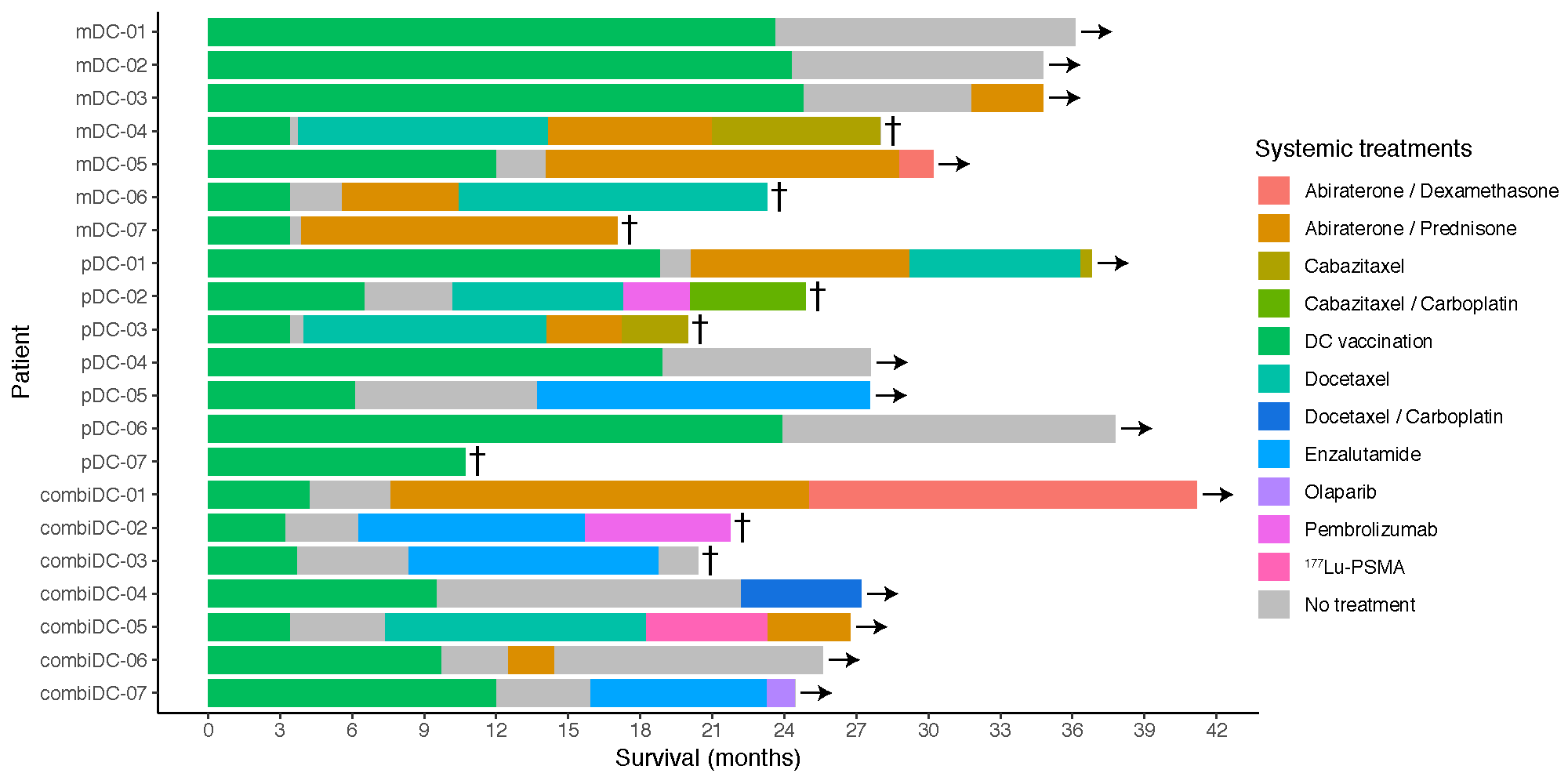

Supplement: Supplementary file 6 — Additional file 6: Figure S6. Patient response, survival and systemic treatments since start of DC vaccination. Swimmer plot showing long-term clinical course for each patient. An arrow indicates that the patient is alive at last follow-up. A cross indicates patient demise. [file 40425_2019_787_MOESM6_ESM.tiff]

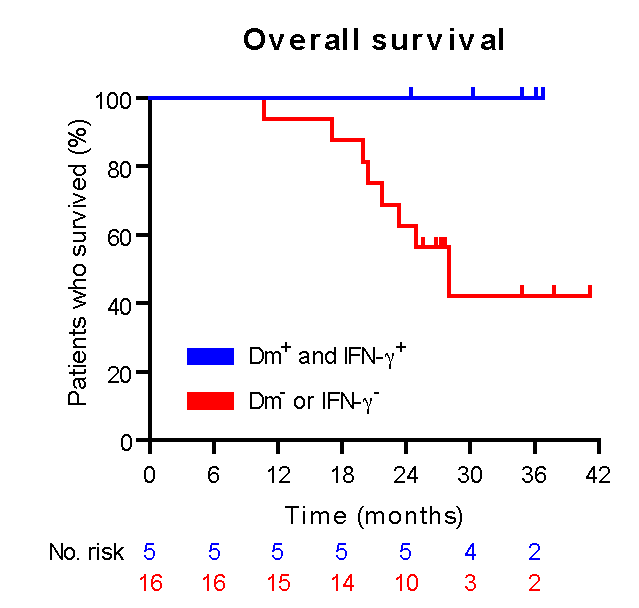

Supplement: Supplementary file 7 — Additional file 7: Figure S7. Kaplan-Meier estimates of survival. Kaplan-Meier analysis of overall survival of patients with (dm+ and IFN-y+) or without (dm− or IFN-y−) the presence of functional antigen-specific T cells in skin biopsies. [file 40425_2019_787_MOESM7_ESM.tiff]
