# Supplementary material for: Blood-derived dendritic cell vaccinations induce immune responses that correlate with clinical outcome in patients with chemo-naive castration-resistant prostate cancer
Source: J Immunother Cancer. 2019 Nov 14;7:302. doi: 10.1186/s40425-019-0787-6 (PMC6854814; doi:10.1186/s40425-019-0787-6)
Supplement: Supplementary file 8 — Additional file 8: Table S1. Immunological, immunohistochemical and whole genome sequencing data. [file 40425_2019_787_MOESM8_ESM.docx]

| Blood-derived DC treated patient^~^ | NY-ESO-1 specific SKILs | MAGE-C2 specific SKILs | MUC1 specific SKILs | NY-ESO-1 expression^^^  (%) | MAGE-C2 expression^^^  (%) | MUC1 expression^^^  (%) | IFN-γ^+^ SKILs | NY-ESO-1 expression  post-vac (%)^()^ | MAGE-C2 expression post-vac (%)^()^ | MUC1  expression post-vac (%)^()^ | PD-L1  expression post-vac (%)^()^ | TML^&^ | Microsatellite status* |
| --- | --- | --- | --- | --- | --- | --- | --- | --- | --- | --- | --- | --- | --- |
| mDC-01  mDC-02  mDC-03  mDC-04  mDC-05  mDC-06  mDC-07  pDC-01  pDC-02  pDC-03  pDC-04  pDC-05  pDC-06  pDC-07  combiDC-01  combiDC-02  combiDC-03  combiDC-04  combiDC-05  combiDC-06  combiDC-07 | +  +  +  +  +  - +  + -  - +  - + + - +  + + - +  + | +  +  +  +  -  - -  - -  - +  - + + - +  + + - +  + | -  -  +  -  -  - -  - -  - +  - - + - -  - + - +  - | 0  0  0  0  0  na  0  0  na  0  0  0  0  0  0  0  0  40  na  0  0 | 0  0  0  0  0  na  20  0  na  10  5  60  0  0  0  0  0  0  na  0  0 | 1  10  5  <1  0  na  100  0  na  0  0  5  25  5  <1  30  1  90  na  10  10 | +  +  -  -  +  + -  + +  - -  + - - - -  - - - -  + | 0  na  na  na  na  0  na  na  0  na na  na  na  na  na  na  <1  na  0 0  na | 0  na  na  na  na  0  na  na  0  na na  na  na  na  na  na  <1  na  0 0  na | 0  na  na  na  na  10  na  na  10  na na  na  na  na  na  na  2  na  0 0  na | 0  60  na  0  0  na na  na 5  <1 na  0 na na na 0  na na na 0  0 | 28  na  na  25  na  47 na  na 56  46 na  na na na na 105  26 na 11 34  74 | MSS  MSS  na  MSS  na  na  na  na  MSS  MSS  na  MSS  na  na  na  MSS  MSS  na  MSS  MSS  MSS |

**Additional file 8: Table S1. Immunological, immunohistochemical and whole genome sequencing data.**

DC: dendritic cells; Dm: dextramer; LN: lymph nodes; MSI: microsatellite instable; MSS: microsatellite stable; na: not available; post-vac: post-vaccination; SKILs: skin-infiltrating lymphocytes; TML: tumor mutational load.

^ immunohistochemical staining for NY-ESO-1, MAGE-C2 and MUC1 performed on primary prostate biopsies, prostatectomy tissue or lymph nodes metastases all sampled prior to the start of DC vaccinations.

() immunohistochemical staining for NY-ESO-1, MAGE-C2, MUC1 and PD-L1 performed on lymph nodes metastases or bone metastases all sampled after progression on DC vaccination therapy.

& microsatellite stability score represents the number of somatic inserts and deletions in short repeat sections across the whole genome of the tumor per megabyte.

* tumor mutational load represents the total number of somatic missense variants across the whole genome of the tumor.
